# Ionic species programmable synaptic plasticity in multimodal nanofluidic devices

**DOI:** 10.1093/nsr/nwag036

**Published:** 2026-01-19

**Authors:** Miliang Zhang, Ronghua Lan, Zhixiao Si, Jiqing Dai, Wenchao Liu, Wenbo Chang, Junjun Liu, Guoheng Xu, Kai Xiao

**Affiliations:** School of Materials and Environmental Engineering, Shenzhen Polytechnic University, Shenzhen 518055, China; Department of Biomedical Engineering, Guangdong Provincial Key Laboratory of Advanced Biomaterials, Institute of Innovative Materials, Southern University of Science and Technology (SUSTech), Shenzhen 518055, China; School of Materials and Environmental Engineering, Shenzhen Polytechnic University, Shenzhen 518055, China; Department of Biomedical Engineering, Guangdong Provincial Key Laboratory of Advanced Biomaterials, Institute of Innovative Materials, Southern University of Science and Technology (SUSTech), Shenzhen 518055, China; Department of Biomedical Engineering, Guangdong Provincial Key Laboratory of Advanced Biomaterials, Institute of Innovative Materials, Southern University of Science and Technology (SUSTech), Shenzhen 518055, China; Department of Biomedical Engineering, Guangdong Provincial Key Laboratory of Advanced Biomaterials, Institute of Innovative Materials, Southern University of Science and Technology (SUSTech), Shenzhen 518055, China; Department of Biomedical Engineering, Guangdong Provincial Key Laboratory of Advanced Biomaterials, Institute of Innovative Materials, Southern University of Science and Technology (SUSTech), Shenzhen 518055, China; Department of Biomedical Engineering, Guangdong Provincial Key Laboratory of Advanced Biomaterials, Institute of Innovative Materials, Southern University of Science and Technology (SUSTech), Shenzhen 518055, China; School of Materials and Environmental Engineering, Shenzhen Polytechnic University, Shenzhen 518055, China; Department of Biomedical Engineering, Guangdong Provincial Key Laboratory of Advanced Biomaterials, Institute of Innovative Materials, Southern University of Science and Technology (SUSTech), Shenzhen 518055, China; Department of Biomedical Engineering, Guangdong Provincial Key Laboratory of Advanced Biomaterials, Institute of Innovative Materials, Southern University of Science and Technology (SUSTech), Shenzhen 518055, China

**Keywords:** nanofluidic iontronics, nanofluidic memristor, nanofluidic capacitor, artificial synapse, neuromorphic device

## Abstract

Nanofluidic devices have been widely utilized to simulate electronic functionalities recently, due to their unique ion transport behaviors, such as non-linear ion transport, selectivity etc. However, the correlation between the ion transport behavior and the transitions among various nanofluidic capacitive and inductive hysteresis still remains poorly understood, which impedes the development of nanofluidic systems. Here, we report a concentration-dependent transition between capacitive and inductive hysteresis in gold-nanoparticle-stacked nanochannels. Quantitative analysis reveals that this transition is governed by the interionic distance relative to the Bjerrum length, establishing a universal mechanism for ion transport modulation. Notably, our system enables unidirectional plasticity (both facilitation and depression) by simply altering the ionic species, demonstrating programmable plasticity without structural reconfiguration. Additionally, a high-pass filter (HPF) circuit with tunable cut-off frequency is implemented through two identical nanofluidic devices. These findings establish a new paradigm for multifunctional nanofluidic devices and provide a rational foundation for the design of aqueous-phase neuromorphic computing circuits.

## Teaser

A single nanofluidic device made of gold-nanoparticle-stacked channels can be reprogrammed with the electrolyte: concentration flips its electrical response, and ion species selects synapse-like facilitation or depression for tunable ionic high pass filter circuits.

## INTRODUCTION

Nanofluidics, the field interested in the transport of fluids and ionic species at nanometric scales [1], has grown at a fast pace. Under confinement, the surface and interface interactions exert paramount influences on the transport and interactive behaviors of ions and molecules, resulting in various anomalous transport behaviors, such as non-linear ion transport [2–4], enhanced water permeability [5] and superior selectivity among similar-sized anions [6]. Comprehensive understanding about the mechanisms behind those phenomena has enabled a broad spectrum of applications, including biopolymer sequencing (e.g. DNA, RNA, amino acids, peptides and proteins) [7–12], osmotic energy harvesting [13,14], biochemical sensing [15–17] and nanofluidic ionic circuits (resistors, capacitors, diodes, transistors, memristors etc. as building blocks) [18–27].

Designing advanced ionic circuits is a challenging quest and is also a promising routine due to their superior biocompatibility and efficient bidirectional information transfer when integrated with biological platforms compared to conventional electron-based systems with rigid, non-deformable planar architectures [28,29]. Although considerable progress has been made in the study of nanofluidic devices, most reported systems are restricted to exhibiting the functionality of a single electronic component, as mentioned above. Devices capable of integrating multiple electronic functionalities—such as diverse capacitive and inductive hysteresis originating from various effects (including electrical double layer (EDL) effect, ion pairing effect, adsorption effect and concentration-polarization (CP) effect)—within a single architecture are exceedingly rare. Even rarer is the realization of complex functions through multiple identical devices, which can significantly reduce circuit complexity and lower fabrication costs. Their working principles are still unclear, which limits their development potential. Meanwhile, exploiting ion species diversity to broaden the functional landscape of nanofluidic devices remains a critical yet insufficiently explored research area, which is significant to the implementation of complex ionic circuits.

Here, we realize confinement space by *in situ* electroless-deposited Au nanoparticles (Au NPs) in polycarbonate track-etch (PCTE) membranes and observe an electrical transition behavior between capacitive and inductive hysteresis dependent on the ionic states, which are highly associated with the relationship between the interionic distance (ionic concentration) and the Bjerrum length [30,31]. At relatively low ionic concentration (large interionic distance), ions are prone to dissociate from each other as point charges (capacitive curve), whereas ions are prone to associate into Bjerrum pairs at high ionic concentration (inductive curve). Our system inherently combines the EDL effect (capacitive hysteresis) and the ion pairing effect (inductive hysteresis) due to strong confinement. Thus, when we say that our device exhibits capacitive characteristics, we mean the capacitive contribution predominates, and vice versa. By incorporating this physical criterion into a coupled model, we reproduce the observed electrical signatures and elucidate the underlying mechanism. Further, we demonstrate ion-dependent unidirectional plasticity—both potentiation and depression—achieved solely by varying the transported ionic species without altering the device architecture. Leveraging these unique features, we construct a high-pass filter (HPF) circuit with two identical devices in series, whose cut-off frequency can be tuned through the choice of electrolyte. These findings offer key insights into the design of advanced nanofluidic devices and sophisticated ionic circuits.

## RESULTS AND DISCUSSION

### Device design and characterization

**Figure 1.**
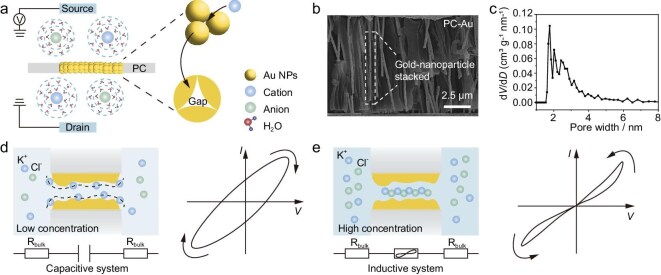
Experimental study of the reversible nanofluidic capacitive and inductive hysteresis transition. (a) Schematic diagram of the electrochemical measurement set-up. Two reservoirs filled with an electrolytic solution are separated by a PCTE membrane deposited with Au NPs. (b) SEM image of the side-view PCTE-Au membrane (150 nm). The white dashed rectangular box represents the nanochannels formed by the stacked Au NPs. Scale bar: 2.5 μm. (c) BET analysis used to determine the size distribution of nanochannels formed by the stacking of Au NPs. (d) The diagram of ionic states at low solution concentration, the equivalent electronic circuit model and the corresponding capacitor *I–V* curve. The capacitance of the nanochannels is around 185 μF. (e) The diagram of ionic states at high solution concentration, the equivalent electronic circuit model and the corresponding inductive *I–V* curve. The resistance of the adjustable resistor ranges from 1.7 kΩ to 28.0 MΩ. Arrows in the figure denote the direction of the applied voltage sweep and the resulting current response. The bulk solution resistance ranges from 14.0 Ω to 26.0 MΩ as calculated.

The experiments were conducted in a homemade two-chamber electrolytic cell separated by a PCTE membrane embedded with *in situ* electroless-deposited Au NPs (PCTE–Au membrane) [32,33]. The chambers were filled with electrolyte solutions and connected to two Ag/AgCl electrodes, as shown in Fig. 1a. Detailed fabrication protocols (Fig. S1), electrochemical measurements, contact angle measurement, scanning electron microscopy (SEM), transmission electron microscopy (TEM), energy-dispersive X-ray spectroscopy (EDS), zeta potential analysis [34], X-ray photoelectron spectroscopy (XPS) and Brunauer–Emmett–Teller (BET) [35] surface area measurements are provided in the Supplementary data and the Methods section. The critical parameter for Au NP growth was deposition time, which directly controlled the pore size of nanochannels [36]. Unless otherwise specified, all electrochemical measurements used 20 nm PCTE–Au membranes (SEM images of pristine 20 nm PCTE membranes are shown in Fig. S6), prepared by 6-h immersion in a Au-plating bath to achieve densely stacked Au NPs in nanochannels. The surface Au was then wiped off with methanol-saturated cotton swabs.

Given the challenges in real-time monitoring of Au deposition within 20 nm PCTE membranes, we employed 150 and 100 nm PCTE membranes as model systems to characterize Au NPs’ stacking dynamics (Fig. S9). The nucleation and growth kinetics of Au NPs were systematically investigated via SEM and TEM across plating times ranging from 1 to 24 h (Fig. S9). These analyses confirm that Au deposition initiates via redox reactions between Au^+^ ions and Ag NPs, forming small nuclei that coalesce over time—a mechanism consistent with prior studies [32]. As quantified in Fig. S9c, g and k, the Au layer thickness reached 51 nm after 6 h of deposition. Thus, based on this kinetic profile, selecting a 6-h plating duration is optimal to achieve densely packed nanoparticles and well-defined nanoconfined channels in 20 nm PCTE membranes. After deposition, the PCTE–Au membrane surface is fully covered by a continuous Au layer (Fig. S7a), while interconnected Au NPs occupy the nanochannels (Fig. 1b). The uniform surface distribution of Au NPs was further validated by EDS imaging (Fig. S7b). To corroborate *in situ* growth mechanisms, PCTE–Au (20 nm) membranes were dissolved in CH_2_Cl_2_, revealing Au nanostructures via TEM analysis analogous to those in larger-channel membranes (Fig. S8a). Particle size histogram analysis (Fig. S8b) indicated a Gaussian distribution centered at 20.5 ± 2.1 nm. The nanochannel size distributions were calculated from CO_2_ adsorption isotherms using the non-local density functional theory (NLDFT) method, with a dominant peak at 1.8 nm (Fig. 1c), which is comparable to the size of mobile ions used in this experiment (Table S2). In this scenario, the surface effect becomes dominant and contributes to the observed exotic phenomena. The main electrical features of this system are presented in Fig. 1d and e: apparent non-linear ion transport, capacitive hysteresis in low concentration electrolyte solution, and inductive hysteresis in high concentration electrolyte solution. Further analyses are presented in subsequent sections.

### Nanofluidic capacitive and inductive hysteresis transition in KCl solution

**Figure 2.**
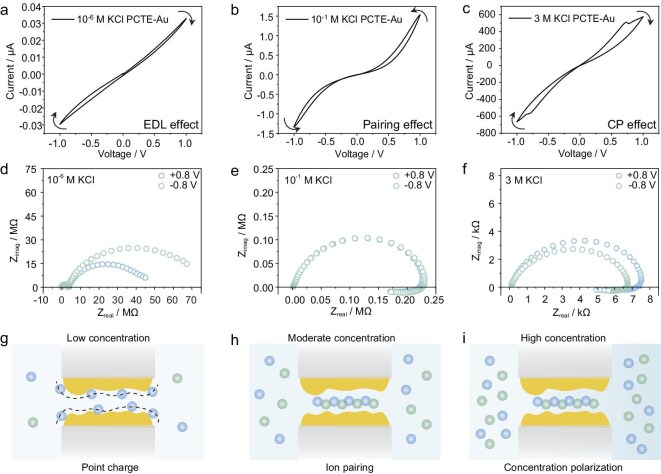
The mechanism and electrical signal of ion transport while using KCl as the electrolyte. (a–c) Representative *I–V* curves for 20 nm PCTE membrane electroless-deposited with Au NPs in (a) 10^−6^ M KCl, (b) 10^−1^ M KCl and (c) 3 M KCl, respectively. (d–f) EIS spectra of 20 nm PCTE membrane electroless-deposited with Au NPs in (d) 10^−6^ M KCl, (e) 10^−1^ M KCl and (f) 3 M KCl, respectively (Nyquist plots). (g–i) The ionic states in different concentrations. (g) Low concentration: point charge; (h) moderate concentration: ion pairing; (i) high concentration: ion pairing with concentration polarization. Arrows in the figure denote the direction of the applied voltage sweep and the resulting current response.

Beyond nanochannel size, surface charge polarity and density also critically influence ion transport. Chloride ions (Cl^−^) are known to adsorb onto Au surfaces (Fig. S33), imparting a negative surface potential to Au NPs [37,38]. This surface property is essential in explaining the electrical signals generated by ionic dynamics in our system. According to Fig. 2a–c, the device exhibits a concentration-dependent transition from capacitive to inductive hysteresis, as revealed by the *I*–*V* curves. At low concentrations, the system behaves as an EDL capacitor (EDLC), where ions accumulate onto the Au NP surface [26,39,40]. At higher concentrations, the *I*–*V* response shifts to inductive hysteresis, consistent with the Wien effect, where high electric field induces ion dissociation and enhanced conductivity [2,41]. The unipolar behavior (Fig. 2b), characterized by conductance increasing with bias at both polarities, supports this mechanism. Moreover, the contact angle (CA) measurements (Fig. S11) exclude hydrophobic effects as the origin of this non-linearity [42].

The above observations suggest the existence of two distinct ionic states determined by the relationship between interionic distance ($l$) (Equation 1) and Bjerrum length (*l*_B_) ( Equation 2) [30,31]:


(1) \begin{equation*}l \approx {\left( {\frac{1}{{2c{N}_{\rm A}}}} \right)}^{1/3},\end{equation*}

(2) \begin{equation*}{l}_{\rm B} = \frac{{{e}^2}}{{4\pi {\varepsilon }_0{\varepsilon }_{\rm r}{k}_{\rm B}T}},\end{equation*}

where *c* is the solution concentration and *N*_A_ is the Avogadro constant, *e* is the elementary charge, ε_r_ is the relative dielectric constant of the medium, *ε*_0_ is the vacuum permittivity, *k*_B_ is the Boltzmann constant, and *T* is the absolute temperature in Kelvin. At low ionic strength (Fig. 2g), ions behave as isolated point charges, producing classical capacitive behavior, confirmed by electrochemical impedance spectroscopy (EIS) and semi-circular Nyquist plots [4,43,44]. As concentration increases, ion pairing and clustering occur (Fig. 2h and i). Under applied bias, these ion pairs dissociate into free ions, generating excess conductivity and resulting in the observed non-linear *I*–*V* response [2]. The arc in the fourth quadrant of the EIS spectra (Fig. 2e and f) supports the presence of chemical inductor behavior, which generally arises from two coupled processes. The first process responds rapidly to the external stimulus, and the second process is slow and delayed compared to the first one. In our scenario, it is the ion pairing/dissociation process serving as the second process and the time constant is greater than those of other components in our system (the origin for the chemical inductor behavior) [44–46]. At sufficiently high currents, concentration polarization emerges, leading to the current decrease with an increasing applied voltage (Fig. 2c), frequently observed in PCTE membranes at high electrolyte concentrations (Figs S12, S14, S18, S20, S22 and S24) [47,48]. Prior to the threshold current, PCTE membranes exhibit ohmic behavior, functioning as simple resistors (Figs S12, S14, S18, S20, S22 and S24). The chemical inductor behavior (Fig. 2f) occurs because of relatively short test time when there is no concentration polarization.

All typical *I–V* curves recorded in KCl solution of different concentrations for PCTE–Au membranes are summarized in Fig. S13. A clear transition from the EDL effect and the ion pairing effect to the CP effect is observed while there is not a strong EDL effect at low concentrations for PCTE membranes, highlighting the critical role of the Au NP nanochannels. In contrast to the data for PCTE membranes, the current for PCTE–Au membranes is significantly reduced due to the decreased size of nanochannels (Figs S12 and S13). Notably, the capacitive response remains stable at concentrations below 10^−3^ M, suggesting that capacitance is predominantly governed by surface properties under dilute conditions.

### Simulation of capacitive and inductive hysteresis transition

**Figure 3.**
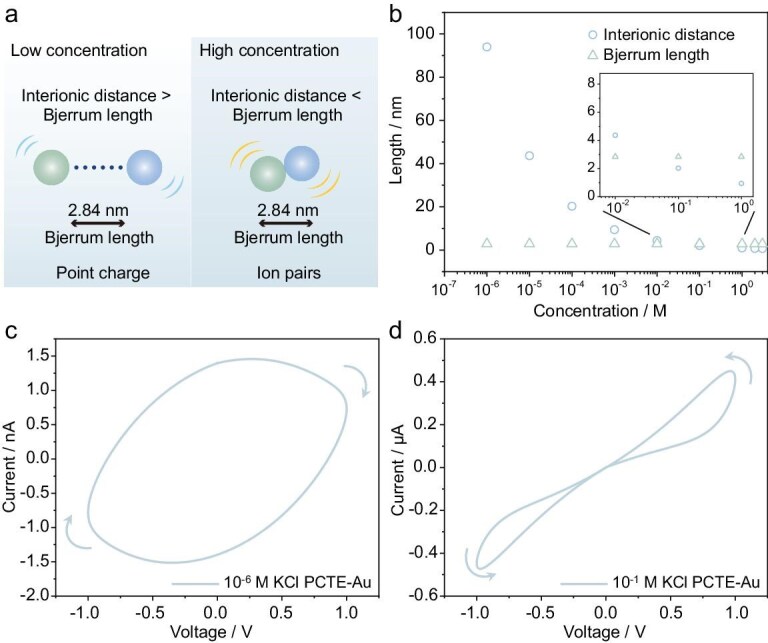
The mechanism and the simulation of the combined system with EDL effect and ion pairing effect. (a) The key parameters determining the ionic states at different solution concentrations. (b) The comparison between interionic distance and Bjerrum length with varying solution concentrations. (c and d) Representative simulated *I–V* curves for (c) 10^−6^ M KCl and (d) 10^−1^ M KCl. Arrows in the figure denote the direction of the applied voltage sweep and the resulting current response.

Based on the above discussion, we are able to propose a comprehensive framework to elucidate how ionic states (determined by the relationship between interionic distance and Bjerrum length, as shown in Fig. 3a and b) under confinement influence the *I–V* response of the system. Our system generally consists of three parts: the capacitance on the electrodes; the bulk solution; and the nanochannels within the membrane. The dramatic transition mainly happens in the nanochannels.

At a low solution concentration, the EDLs overlap within nanochannels and ions are likely to adsorb onto the Au surface, which effectively mimics a parallel-plate capacitor (store energy through charge accumulation). The analytical derivation of the current–voltage relationship for our system (a single capacitor and two resistors connected in series) is presented as follows:


(3) \begin{equation*}{Z}_{\rm c} = \frac{{V( t)}}{{I( t)}} = \frac{1}{{j\omega {C}_{\rm e}}},\end{equation*}

(4) \begin{eqnarray*}\!\!{Z}_{{\mathrm{total}}} = 2{R}_{{\mathrm{bulk}}} + \frac{1}{{j\omega {C}_{\rm e}}} = 2{R}_{{\mathrm{bulk}}} - j\left( {\frac{1}{{\omega {C}_{\rm e}}}} \right)\!,\,\,\end{eqnarray*}

(5) \begin{eqnarray*}
I ( t ) &=& \frac{{{V}_0\sin ( {\omega t} )}}{{\left| {{Z}_{{\mathrm{total}}}} \right|}} = \frac{{{V}_0}}{{\sqrt {{{\left( {2{R}_{{\mathrm{bulk}}}} \right)}}^2 + {{\left( {\frac{1}{{\omega {C}_{\rm e}}}} \right)}}^2} }}\nonumber\\
&&\qquad \quad \times\, \sin \left( {\omega t - \phi } \right),
\end{eqnarray*}

where *Z*_c_ is the impedance of the capacitor system, *V*(*t*) is the applied sinusoidal voltage, *I*(*t*) is the output ionic current, *C*_e_ is the capacitance of the system, *ω* is the angular frequency of the applied voltage, *Z*_total_ is the total impedance of the system, *R*_bulk_ is the bulk solution resistance, *V*_0_ is the amplitude of the applied voltage, and *ϕ* is the phase shift.

At a high solution concentration, ions tend to form Bjerrum pairs under strong confinement, which provides the ion pairing effect in our system. As a result, the system’s output ionic current is a strong non-linear function of voltage, behaving as:


(6) \begin{equation*}I( t) = {G}_0w{( t)}^pV( t),\end{equation*}

where *V*(*t*) is the applied sinusoidal voltage, *G*_0_ is the conductance of ions that are already free at thermal equilibrium (obtained from data in Fig. S13), *w*(*t*) is the internal state variable, *p* is the exponent for conductance non-linearity, and *I*(*t*) is the output ionic current. Thus, we propose qualitatively that two different components coexist in our system and the ratio of them is determined by the solution concentration. We are then able to introduce a concentration-dependent weighting function *Z*(*c*) to quantitatively analyze the system’s *I–V* response as follows [2]:


(7) \begin{eqnarray*}
I (t) &=& \left( {1 - Z\left( c \right)} \right)\frac{{{V}_0}}{{\sqrt {{{\left( {2{R}_{{\mathrm{bulk}}}} \right)}}^2 + {{\left( {\frac{1}{{\omega {C}_{\rm e}}}} \right)}}^2} }}\nonumber\\
&&\times\, \sin ( {\omega t - \phi }) + Z( c){G}_0w{( t)}^pV( t),\nonumber\\
\end{eqnarray*}

The detailed analysis is included in the Supplementary data. The resulting simulation (Fig. 3 and Fig. S17) is in good agreement with experimental data (Fig. 2 and Fig. S13). According to this analysis, a qualitative and quantitative rationalization of the nanofluidic capacitive and inductive hysteresis transition is established, which paves the way for the implementation of other complex ionic circuits.

### Diverse hysteresis with different working carriers

**Figure 4.**
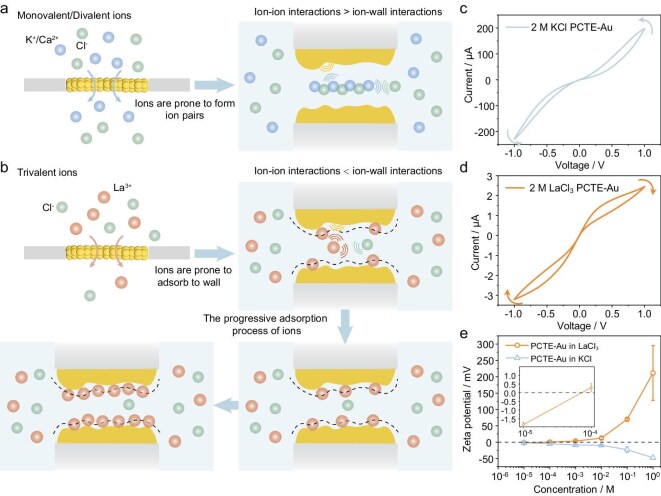
The performance comparison of ion transport with KCl and LaCl_3_ and the origin of the transport behavior. (a and b) Schematic diagram of the ion transport behavior in (a) KCl/CaCl_2_ solution and (b) LaCl_3_ solution. (c) *I–V* characteristics of nanofluidic systems in 2 M KCl. (d) *I–V* characteristics of nanofluidic systems in 2 M LaCl_3_. (e) The zeta potential of PCTE–Au in KCl and LaCl_3_ solutions of different concentrations. Arrows in the figure denote the direction of the applied voltage sweep and the resulting current response.

Following the investigation of ion transport behavior in KCl solutions across varying concentrations, we further examined the role of cation valence and size by replacing K^+^ with other cations to show how ion–wall interactions within confined nanochannels influence transport characteristics (Fig. 4a and b). While monovalent and divalent electrolyte solutions including NaCl, CaCl_2_ and BaCl_2_ (Figs S15, S19 and S21) display a transition behavior similar to KCl (Fig. S13), the performance of trivalent solutions, for example, LaCl_3_ and AlCl_3_ solutions (Fig. 4d and Fig. S25), diverge significantly from that of KCl (Fig. 4c). As shown in Fig. 4c and d, at the same concentrations, the current amplitude in KCl is notably higher than in LaCl_3_. Moreover, KCl exhibits increasing conductance with applied voltage, characteristic of the ion pairing effect, whereas LaCl_3_ shows decreasing conductance, indicating fundamentally different transport mechanisms. To figure out the mechanism behind this, we conduct zeta potential measurement (the surface Au layer of PCTE–Au membranes for the test was retained). As shown in Fig. 4e, the zeta potential for PCTE–Au membranes in KCl remains negative across all concentrations (from 10^−5^ to 1 M) and gradually becomes more negative as concentration increases. In LaCl_3_ solutions (Fig. 4e), the zeta potential of the PCTE–Au membrane remains negative at low concentrations (10^−5^ M), indicating a negatively charged surface. As the LaCl_3_ concentration increases, the zeta potential increases significantly and eventually becomes positive, demonstrating a clear charge inversion phenomenon (Fig. 4b) [49,50]. Furthermore, we can substantially prove the adsorption behavior of La^3+^ from the high-resolution XPS spectra for PCTE–Au membranes in four different solutions (purified water; KCl solution; LaCl_3_ solution; an equal-volume (1:1, v/v) mixture of KCl and LaCl_3_ solutions) (Figs S31–35). In this circumstance, the ion–ion interactions are smaller than ion–wall interactions, which leads to the progressive adsorption of La^3+^. This adsorption of La^3+^ not only results in the surface charge inversion upon reaching a certain threshold but also causes the blockage of nanochannels, especially when the size of La^3+^ is comparable to the size of the nanochannels [50]. These synergistic effects (charge density, charge polarity and effective radius) significantly reduce ionic conductance.

### Programmable HPF construction by diverse hysteresis (EDL effect, ion pairing effect and adsorption effect)

**Figure 5.**
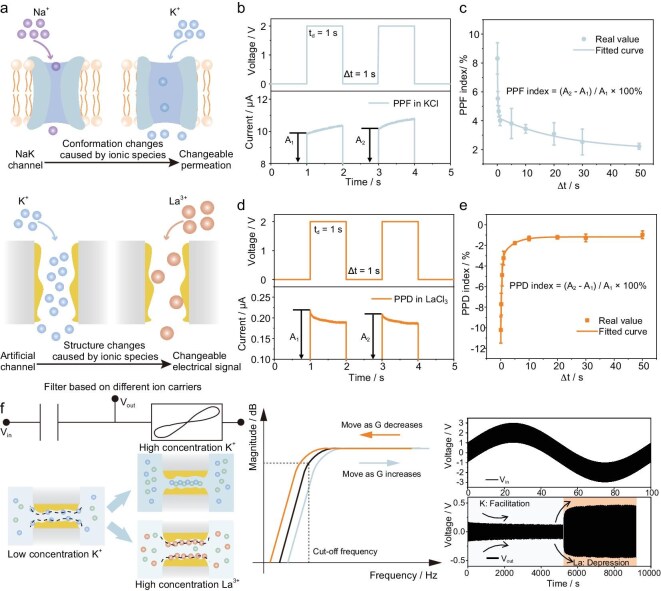
The distinct electrical signal while using different ions as carriers and the HPF circuit. (a) Schematic comparison between the biological NaK channel and the artificial channel (PCTE–Au), both of which exhibit different electrical outputs depending on the ionic species transported through the same nanochannel structure. (b and c) Realization of PPF and corresponding PPF index when KCl is used as the electrolyte. (d and e) Realization of PPD and corresponding PPD index when LaCl_3_ is used as the electrolyte. (f) An HPF circuit realized through two identical nanofluidic devices with distinct hysteresis (EDL effect, ion pairing effect and adsorption effect) in the serial configuration.

Considering the La^3+^ adsorption behavior and distinct electrical signals, the system emulates specific functions of NaK channels via the Au-NP-stacked nanochannels [51]. It is reported that the ion binding will induce the change of NaK channel conformation, which will further alter its ion selectivity and permeability (Fig. 5a). In our system, the ion pairing (1 M KCl) and ion adsorption (1 M LaCl_3_) behaviors are utilized to realize unidirectional plasticity, including paired-pulse facilitation (PPF) and paired-pulse depression (PPD). For the KCl solution, two sequential voltage pulses (*V* = 1 V, Δ*t* = 1 s) are applied to PCTE–Au membranes in the configuration, as in Fig. 1a. The currents induced by the second stimulus (*A*_2_) are greater than those induced by the first stimulus (*A*_1_) (Fig. 5b). For the LaCl_3_ solution, the trends are completely reversed, as shown in Fig. 5d. The PPF and PPD indices, both defined as (*A*_2_ *−* *A*_1_)/*A*_1_ × 100%, are correlated with the interval time (Δ*t*) between the paired pulses, decaying biexponentially as Δ*t* increased (Fig. 5c and e). The PPF and PPD indices can be expressed using the following formulas:


(8) \begin{equation*}{\mathrm{PPF}}\ \textit{index} = {C}_1 \times \exp \left(\! { - \frac{{\Delta t}}{{{\tau }_1}}} \right) + {C}_2 \times \exp \left(\! { - \frac{{\Delta t}}{{{\tau }_2}}} \right),\end{equation*}

(9) \begin{equation*}{\mathrm{PPD}}\ \textit{index} = {C}_3 \times \exp \left(\! { - \frac{{\Delta t}}{{{\tau }_3}}} \right) + {C}_4 \times \exp \left(\! { - \frac{{\Delta t}}{{{\tau }_4}}} \right),\end{equation*}

where *C*_1_, *C*_2_, *C*_3_ and *C*_4_ are constant, Δ*t* is the time interval between two pulses, and *τ*_1_, *τ*_2_, *τ*_3_ and *τ*_4_ are relaxation time constants, which well reflect the kinetic process of ion pairing and adsorption/desorption (as shown in the Supplementary data). This property can extend the potential applications of nanofluidic devices for multiple functionalities.

In addition to modulating the performance by altering ion species, we also make efforts to modify the surface of Au NPs with cysteamine. The cysteamine modification not only makes the surface charge more positive but also reduces the effective radius of the nanochannels, which significantly changes the capacitance and conductance of our system, and these variations further influence the shape and current of the *I–V* curve, as shown in Fig. S30.

Building on the diverse hysteresis (originating from EDL effect, ion pairing effect and adsorption effect) of our devices mentioned above, we next implement an HPF circuit by connecting two identical devices in series (one serving as the capacitor and the other serving as the adjustable resistor due to varying ionic states) (Fig. 5f) [52]. The nanofluidic capacitors are realized by employing a low-concentration KCl solution as the electrolyte, whereas a high-concentration KCl or LaCl_3_ solution is used to achieve the variable resistance. Applying input voltage to the HPF circuit, the corresponding transfer function and the cut-off frequency can be obtained as follows (the detailed derivations are provided in the Supplementary data):


(10) \begin{equation*}
H\left( {j\omega } \right) = \frac{{{V}_{\rm out}}}{{{V}_{\rm in}}} = \frac{1}{{1 + \frac{1}{{j\omega {R}_{\rm M}C}}}},\end{equation*}

(11) \begin{equation*}f = \frac{1}{{2\pi {R}_{\rm M}C}},\end{equation*}

where *C* is the capacitance of the nanofluidic capacitor, *R*_M_ is the variable resistance, *V*_in_ is the input voltage, *V*_out_ is the output voltage, *H*(*jω*) is the transfer function, and *f* is the cut-off frequency. The cut-off frequency is strongly dependent on *R*_M_, which varies with ionic species under a constant input voltage, thereby enabling dynamic control over the filtering characteristics. To demonstrate this functionality, we apply a hybrid frequency input voltage to the HPF circuit as follows:


(12) \begin{equation*}{V}_{\rm in} (t) = 2 {\rm sin} ( {2\pi \cdot 0.01 \cdot t}) + {\rm sin} ( {2\pi \cdot 120 \cdot t} ).\end{equation*}

When KCl solution was used as an electrolyte, the amplitude of the high-frequency output signal initially decreased due to the increasing conductance (Fig. 5f). Upon replacing the KCl solution with LaCl_3_ solution, a gradually increased amplitude of the high-frequency output signal is realized through depression behavior of the LaCl_3_ solution (Fig. 5f). The detailed amplitude variations are provided in the Supplementary data. The dynamically controllable HPF circuit not only extends the design paradigm of ionic circuits but also highlights the functional diversity and real-time adjustability of nanofluidic devices enabled by the ions’ selection and ionic states. This validation may pave the way for broader implementation of nanofluidic circuits in diverse application scenarios.

## CONCLUSION

In summary, we present a bottom-up nanochannel fabrication strategy that exhibits strong confinement effects. The channel size and surface properties can be precisely modulated by adjusting plating time and employing diverse post-modification approaches. Within this system, a reversible transition between capacitive and inductive hysteresis is achieved solely by varying the solution concentration. This phenomenon is observed for low-valency ions (e.g. K^+^, Na^+^, Ca^2+^, Ba^2+^) and is closely associated with ionic states, as confirmed by EIS analysis. In particular, a systematic model is used to demonstrate how solution concentration changes ionic states and influences the *I–V* curve transition. Our work also illustrates how La^3+^ adsorption is harnessed to realize tunable plasticity from PPF (KCl solution) to PPD in the same device platform. Furthermore, we realized a dynamically controllable HPF circuit and tunable plasticity (PPF and PPD). Our system cannot currently match the performance of solid-state devices and circuits, but it offers novel and versatile design strategies for multifunctional nanofluidic devices and ionic circuits. Further developments, such as high integration density, long-term stability and high switching speed enabled by advanced fabrication and optimized designs, will help us construct the fully liquid circuits like the brain of living organisms.

## MATERIALS AND METHODS

### Materials

PCTE filtration membranes (Whatman, 47 mm filter diameter, 7–22 µm filter thickness) with different nominal pore diameters of 20, 100 and 150 nm, coated by the producer with wetting agent polyvinylpyrrolidone (PVP), were used as the template to prepare the PCTE–Au membrane. Two Ag/AgCl wires were used as electrodes. Cysteamine, methanol, formaldehyde solution, tin chloride, trifluoroacetic acid, ammonia solution, KCl, CaCl_2_ and LaCl_3_ heptahydrate were from Aladdin. Sodium gold sulfide [Na_3_Au(SO_3_)_2_] was from Weng Jiang Reagent Co. Dichloromethane was from J&K Scientific. Silver nitrate was from Xilong Scientific. Sodium sulfite was from MREDA. Nitric acid was from Shanxi Xihua Chemical Industry Co., Ltd. Purified water (∼18.2 MΩ·cm) was obtained using a Milli-Q (Millipore) water purification system. All chemicals were used without further purification.

### Preparation of PCTE–Au membranes and PCTE–Au–NH_2_ membranes

The PCTE membrane is a kind of frequently used membrane in template synthesis. Here we use commercial PCTE membranes as our template. The fabrication process generally involves three steps, as shown in Fig. S1. First, heavy ions are used to strike the membrane surface, facilitating the formation of latent tracks as they travel through the membrane. Second, the open-ended pores are formed by the chemical etching of the latent tracks. Finally, the membranes are made hydrophilic via PVP solution immersion, which is helpful for the adsorption of Sn^2+^.

The PCTE–Au membranes are fabricated following the reported procedures (Fig. S1). Following a 2-h pre-wetting process in methanol, the PCTE template membranes underwent sensitization with Sn^2+^ by immersion in a solution composed of 0.026 M SnCl_2_ and 0.07 M trifluoroacetic acid in a 50:50 methanol–water mixture for 1 h. The membrane was then rinsed with methanol for 5 min before being exposed to a 0.029 M Ag[(NH_3_)_2_]NO_3_ solution for 1 h to facilitate Ag deposition. Following this step, the membrane was immersed in an Au plating bath containing 0.625 M formaldehyde, 0.127 M Na_2_SO_3_ and 7.9 × 10^−3^ M Na_3_Au(SO_3_)_2_. The temperature was maintained in 4°C. The Ag particles are gradually displaced by Au NPs since Au is a more noble metal. As a result, we obtain an Au-NP-stacked PCTE membrane with different filling levels (varying the time for Au deposition). Without further statement, all PCTE–Au membranes used for electrochemical measurement are 20 nm PCTE membranes (Fig. S6) deposited with Au NPs for 6 h. The outer faces of the membrane were cleaned with Scotch tape and/or cleaned with Q-tips embedded with methanol. The other PCTE–Au membranes (100 and 150 nm) are used to estimate the growth process since it is difficult to record the Au growth process in 20 nm PCTE membranes.

The PCTE–Au–NH_2_ membranes are fabricated by immersing PCTE–Au membranes in 100 mM cysteamine aqueous solution for 12 h.

## Supplementary Material

nwag036_Supplemental_File
