# Supplementary material for: Ionic species programmable synaptic plasticity in multimodal nanofluidic devices
Source: Natl Sci Rev. 2026 Jan 19;13(5):nwag036. doi: 10.1093/nsr/nwag036 (PMC12976602; doi:10.1093/nsr/nwag036)
Supplement: nwag036_Supplemental_File [file nwag036_supplemental_file.pdf]

Supporting Information for
Ionic Species Programmable Synaptic Plasticity in Multimodal Nanofluidic Devices

Miliang Zhang[1,2], Ronghua Lan[1,2], Zhixiao Si[2], Jiqing Dai[2], Wenchao Liu[2], Wenbo Chang[2], Junjun Liu[1,*], Guoheng Xu[2,*], Kai Xiao[2,*]

*Kai Xiao, Guoheng Xu, Junjun Liu

Emails:
xiaok3@sustech.edu.cn;
guohengxu0706@163.com;
liujunjun@szpu.edu.cn;

This PDF file includes:

Theoretical foundations of numerical simulation;
Theoretical foundations of the high-pass filter circuit based on diverse hysteresis (EDL effect, ion pairing effect and adsorption effect);
Electrochemical measurements;
Material characterization;
BET measurement and NLDFT analysis;
Table S1 to S3;
Figure S1 to S39;
SI References;

Supporting Information Text

Theoretical foundations of numerical simulation

In this system, there are two kinds of ionic states (separated point charges and Bjerrum pairs) coexisting within the gold-nanoparticle-stacked nanochannels, which is highly related with the comparison of interionic distance and Bjerrum length (l_B).

$$l_B = \frac{e^2}{4\pi\varepsilon_0\varepsilon_r k_B T} \quad \text{(S1)}$$

The Bjerrum length is the separation at which the electrostatic interaction between two elementary charges is comparable in the magnitude to the thermal energy scale. Where e is the elementary charge, ε_r is the relative dielectric constant of the medium, ε_0 is the vacuum permittivity, k_B is the Boltzmann constant and T is the absolute temperature in Kelvins. For water at room temperature ($T \approx 298$ K), $\varepsilon_r \approx 80$, so that $l_B \approx 0.71$ nm. It has been proved that the dielectric constant of water under confinement will dramatically change because of the varying rotational freedom of water dipoles (combined in-plane dielectric constant $\varepsilon_{\parallel}$ and out-of-plane dielectric constant $\varepsilon_{\perp}$). According to previous reports, it is reasonable for us to set the relative dielectric constant of the medium to 20 [1, 2]. Then the Bjerrum length in our system is approximately 2.84 nm.

Under the idealized assumption of uniformly distributed hard-sphere ions in a 1:1 electrolyte, the interionic distance (l) equals the cube root of the mean volume occupied by each ion in the solution and we can simply obtain the interionic distance from the equation below: [3]

$$l \approx \left(\frac{1}{2cN_A}\right)^{1/3} \quad \text{(S2)}$$

where c is the ion concentration and N_A is the Avogadro constant (6.022 × 1023 mol^{-1}). According to the above equation, the interionic distance in 0.1 M KCl is approximately 2.02 nm (4.36 nm for 0.01 M KCl) and will become smaller in higher concentration. When the solution concentration is higher than 0.1 M (the interionic distance becomes less than the Bjerrum length), the electrostatic attraction between oppositely charged ions can overcome thermal agitation, leading to the formation of Bjerrum pairs. The Bjerrum pairs dynamics and surface adsorption account for diverse effects and the further *I-V* curve. In contrast, ions are prone to separate with each other and adsorb to the gold surface (working as capacitors) if the solution concentration is less than 0.1 M.

Considering the above situation, we proposed an integrated framework to elucidate how the ionic states under confinement influence the *I-V* response of the system by coupling the EDL effect and ion pairing effect transition model through a concentration-dependent weighting function $z(c)$ as following:

$$z(c) = \frac{1}{1+e^{-\alpha_1(c-\beta_1)}} \quad \text{(S3)}$$

where c is the solution concentration, β_1 describes the transition point (0.1 as discussed above) where the inductive hysteresis dominates in our system and α_1 shows the transition sharpness between point charge and Bjerrum pairs which is set as 34 to have a good simulation of our experiment results.

In relatively low solution concentration, the entire system is represented as a series circuit comprising two bulk solution resistance and a capacitor (Fig. 4). The *I-V* curve can be described as below. For capacitors:

$$I(t) = C_e\frac{dV(t)}{dt} \quad \text{(S4)}$$

$$V(t) = V_0 e^{\omega t j} \quad \text{(S5)}$$

$$Z_c = \frac{V(t)}{I(t)} = \frac{1}{j\omega C_e} \quad \text{(S6)}$$

where $V(t)$ is the applied sinusoidal voltage, V_0 is the amplitude of applied voltage, $I(t)$ is the output ionic current, C_e is the capacitance of the system, ω is the angular frequency of the applied voltage and Z_c is the impedance of the capacitor system. In nanofluidic field, the system capacitance mainly stems from the Helmholtz layer (C_H) and the diffusion layer (C_D), whereas the Helmholtz layer (thickness ranging from 0.3 nm to 0.5 nm) dominates in our system because of the small nanochannels size (around 1.8 nm), as below:

$$\frac{1}{C_e} = \frac{1}{C_H} + \frac{1}{C_D} \quad \text{(S7)}$$

$$C_e \approx C_H \quad \text{(S8)}$$

$$C_H = \frac{\varepsilon_0 \varepsilon_r}{d_H} \quad \text{(S9)}$$

where ε_r is the relative dielectric constant of the medium, ε_0 is the vacuum permittivity and d_H ranges from 0.3 nm to 0.5 nm [4]. For the bulk solution resistance, it can be calculated as follow in different solution concentration: [5]

$$\Lambda(c) = 150.000 - 99.282c^{1/2} + 135.798c - 120.788c^{3/2} + 57.891c^2 - 11.725c^{5/2} \quad \text{(S10)}$$

$$\Lambda(c) = \frac{\kappa}{c} \quad \text{(S11)}$$

$$R_{bulk} = \frac{L}{A\kappa} \quad \text{(S12)}$$

where c is the solution concentration, $\Lambda(c)$ is the molar conductivity, κ is the conductivity, L is the length of bulk solution (set as 1 cm) and A is the cross-sectional area (0.25 cm^2). Then we can obtain the total impedance of the system:

$$Z_{total} = 2R_{bulk} + \frac{1}{j\omega C_e} = 2R_{bulk} - j\left(\frac{1}{\omega C_e}\right) \quad \text{(S13)}$$

$$|Z_{total}| = \sqrt{(2R_{bulk})^2 + \left(\frac{1}{\omega C_e}\right)^2} \quad \text{(S14)}$$

$$\phi = arctan\left(\frac{1}{2\omega C_e R_{bulk}}\right) \quad \text{(S15)}$$

where ϕ is the phase shift and the corresponding output current is as follow:

$$I(t) = \frac{V_0 \, sin(\omega t)}{|Z_{total}|} = \frac{V_0}{\sqrt{(2R_{bulk})^2 + \left(\frac{1}{\omega C_e}\right)^2}} sin(\omega t - \phi) \quad \text{(S16)}$$

In relatively high solution concentration, the system exhibits typical characteristics of inductive hysteresis behavior for Bjerrum pairs. Its typical *I-V* curve can be described as: [6]

$$V(t) = V_0 \, sin(\omega t) \quad \text{(S17)}$$

$$\frac{dw(t)}{dt} = \alpha_2 |V(t)|^m \big(1 - w(t)\big)^n - \beta_2 w(t) \quad \text{(S18)}$$

$$I(t) = G_0 w(t)^p V(t) \quad \text{(S19)}$$

where $V(t)$ is the applied sinusoidal voltage, V_0 is the amplitude of applied voltage, $I(t)$ is the output ionic current, ω is the angular frequency of the applied voltage, $w(t)$ is internal state variable, α_2 is the positive rate constant, β_2 is the decay constant, m is the voltage nonlinearity exponent, n is the nonlinearity for saturation, G_0 is the conductance of ions that are already free at thermal equilibrium (obtained from data in Fig. S13) and p is the exponent for conductance nonlinearity. The value of the parameters used above is chosen based on the previous works and current experiments data in order to have a good

reflection of experimental conditions [6]. Thus, we are able to establish a unified model that links the interionic distance with the ionic states (including free ions and Bjerrum pairs). These variations, in turn, significantly modulates the effective charge carrier, thereby altering the current-voltage characteristics of the system. By combining equation S3, S16 and S19, we can derive the total output current of the system as below:

$$I(t) = \left(1 - Z(c)\right)\frac{V_0}{\sqrt{(2R_{bulk})^2+\left(\frac{1}{\omega C_e}\right)^2}} sin(\omega t - \phi) + Z(c)G_0 w(t)^p V(t) \quad \text{(S20)}$$

In all cases, the output current was calculated via MATLAB and export as corresponding *I-V* curve in varying solution concentrations, as shown in Fig. S17.

Theoretical foundations of the high-pass filter circuit based on diverse hysteresis (EDL effect, ion pairing effect and adsorption effect)

Filter, invented by Campbell and Wagner [7], is significant in the field of signal processing such as communication engineering, digital image processing, and so on. According to the pass band and stop band, filter can be classified as low-pass filter (LPF), high-pass filter (HPF), band pass filter and band stop filter. A basic HPF can be constructed by connecting a resistor and a capacitor in series, where the capacitor allows high-frequency signals to pass while attenuating low-frequency components. By replacing the ordinary resistor with our devices with varying resistance as shown in Fig. 5, we can explore the time-variation characteristic of the tunable filter circuit. Specifically, the analysis of the transfer function and cut-off frequency is illustrated below.

For the RC circuit:

$$Z_C = \frac{1}{j\omega C} \tag{S21}$$

$$Z_{total} = Z_C + R = \frac{1}{j\omega C} + R \tag{S22}$$

$$V_{out} = \frac{R}{R+\frac{1}{j\omega C}} V_{in} = \frac{j\omega RC}{1+j\omega RC} V_{in} \tag{S23}$$

where C is the capacitance of the capacitor, R is the resistance of the resistor, V_{in} is the input voltage and V_{out} is the resistor voltage. The transfer ratio of resistor voltage and input voltage is as follow:

$$H(j\omega) = \frac{V_{out}}{V_{in}} = \frac{R}{R+\frac{1}{j\omega C}} = \frac{1}{1+\frac{1}{j\omega RC}} \tag{S24}$$

The cut-off frequency is determined by

$$f = \frac{1}{2\pi RC} \tag{S25}$$

Further, the HPF based on the diverse hysteresis (EDL effect, ion pairing effect and adsorption effect) as shown in Fig. 5 is analyzed. The transfer function and the cut-off frequency of the circuit can be obtained as follows:

$$H(j\omega) = \frac{1}{1+\frac{1}{j\omega R_M C}} \tag{S26}$$

$$f = \frac{1}{2\pi R_M C} \tag{S27}$$

where R_M is the variable resistance of our devices (in high concentration solution) under the input voltage. Thus, the filtering capability and cut-off frequency of the circuit are time-varying, as shown in Fig. 5.

According to discussion above, we are able to obtain the output amplitude of each frequency component as follow:

$$V_{\mathrm{out}}(f_t) = V_{\mathrm{in}}(f_t) \cdot \frac{f_t/f}{\sqrt{1+(f_t/f)^2}} \tag{S28}$$

where f_t is the tested output frequency, f is the cut-off frequency. So we can derive the diagram of the cut-off frequency over time based on the above formula, as shown in Fig. S39.

Electrochemical measurements

The current-voltage responses and AC impedance spectra of the PCTE-Au membranes were studied with the sourcemeter (KEITHLEY 2634B system) and the potentiostat Reference 600 from Gamry Inc. In the experimental setup, the electrode spacing was 3.5 cm, and the tested membrane was circular with a diameter of 0.8 cm. In the impedance measurements, the amplitude of the AC waveform was set to 10 mV. The frequency range was in general from 100 mHz to 1 MHz.

Material characterization

The morphology of PCTE membranes and PCTE-Au membranes was characterized by a SEM (ZEISS Sigma 300, Germany) at a 15 kV acceleration voltage. The morphology of Au NPs was characterized by a TEM (JEM 2100). The elemental analysis of Au NPs was performed by an energy-dispersive X-ray spectroscope (ZEISS Sigma 300, Germany). The contact angle of PCTE membranes and PCTE-Au membranes was measured by a Drop Shape Analyzer (KRUss DSA25). The XPS measurements for PCTE-Au membranes in four different solutions were measured by Thermo Kalpha. The size distribution of PCTE-Au membranes was measured by accelerated surface area and porosimetry system (AUTOSORB IQ). The zeta potential of PCTE-Au membranes in different concentration electrolyte solutions (KCl and $LaCl_3$ solutions) was measured by electrokinetic analyzer (Anton Paar surpass 3).

BET measurement and NLDFT analysis

The CO_2 adsorption experiments were collected at 273 K up to 1 bar using a high vacuum physisorption analyzer (Autosorb IQ). Before adsorption analysis, the samples were evacuated at 150 °C overnight using a turbomolecular vacuum pump. The pore size distributions were calculated from CO_2 adsorption isotherms using the non-local density flood theory (NLDFT) method. Helium (99.999%) is used for free space measurements. Ultra-high purity grade CO_2 (99.999%) is used for adsorption measurements. To maintain temperature stability, gas isotherms at 273 K were performed in an ice water bath.

NLDFT based methods for pore size/volume analysis have been included in commercial software and are also featured in international standards (such as ISO 15901-2:2022).

These methods allow one to calculate for a particular adsorptive/adsorbent pair a series of theoretical isotherms, N (p/p^0, W), in pores of different widths for a given pore shape. The series of theoretical isotherms is called the kernel, which can be regarded as a theoretical reference for a given class of adsorbent/adsorptive system. The calculation of the pore size distribution function f (W) is based on a solution of the general adsorption isotherm (GAI) equation, which correlates the experimental adsorption isotherm N (p/p^0) with the kernel of the theoretical adsorption or desorption isotherms N (p/p^0, W). For this purpose, the GAI equation is expressed in the form:

$$N(p/p^{o}) = \int_{W_{\min}}^{W_{\max}} N(p/p^{o}, W) f(W) dW \qquad \text{(S29)}$$

Although the solution of the GAI equation with respect to the pore size distribution function f(W) is strictly an ill-posed numerical problem, it is now generally accepted that meaningful and stable solutions can be obtained by using regularisation algorithms [8-10]. In our experiments, we choose CO_2 at 273 K on carbon (slit pore, NLDFT model) as our referred kernel. The calculated total pore volume is 0.103 cc/g and the surface area is 81.24 m^2/g.

Table S1. Parameters used for the numerical simulation

Name	Descriptions	Value
e	Elementary charge	1.602×10^{-19} C
ε_0	Vacuum permittivity	8.854×10^{-12} F/m
ε_r	The relative dielectric constant of the medium	20 (for the confined water)
k_B	Boltzmann constant	1.381×10^{-23} J/K
T	The absolute temperature in Kelvins	298 K
c	The ion concentration	From 10^{-6} to 3 M
N_A	The Avogadro constant	6.022×10^{23} mol^{-1}
α_1	Constant for weighting function	34
β_1	Constant for weighting function	0.1
V_0	The amplitude of applied voltage	1 V
$V(t)$	The applied sinusoidal voltage	---
$I(t)$	The output ionic current	---
C_e	The capacitance of the system	---
ω	The angular frequency of the applied voltage	$\frac{\pi}{400}$
Z_c	The impedance of the capacitor system	---
C_H	The capacitance of the Helmholtz layer	---
C_D	The capacitance of the diffusion layer	---
d_H	The thickness of the Helmholtz layer	From 0.3 nm to 0.5 nm
$\Lambda(c)$	The molar conductivity	---
κ	The conductivity	---
L	The length of bulk solution	1 cm
A	The cross-sectional area of the bulk solution	0.25 cm^2
R_{bulk}	The resistance of the bulk solution	---
α_2	The positive rate constant	0.01
β_2	The decay constant	0.003
m	The voltage nonlinearity exponent	4

n	The nonlinearity for saturation	1
G_0	The conductance of ions that are already free at thermal equilibrium	35×10^{-9} S, 35×10^{-9} S, 40×10^{-9} S, $50{*}\times10^{-9}$ S, 90×10^{-9} S, 1.5×10^{-6} S,15×10^{-6} S ,200×10^{-6} S, 600×10^{-6} S from 10^{-6} to 3 M, sequentially
p	The exponent for conductance nonlinearity	1

Table S2. The relationship between the pore size of the PCTE-Au membrane and the size of mobile ions in the electrolyte

Ion	Ionic diameter (Å)	Hydrated diameter (Å)
Cl^-	3.6	6.6
Li^+	1.9	7.6
Na^+	2.3	7.2
K^+	3.0	6.6
Cs^+	3.6	6.6
Ca^{2+}	2.5	8.4
Ba^{2+}	2.7	8.08
Mg^{2+}	1.4	8.6
Zn^{2+}	1.5	8.6
Al^{3+}	1.1	9.6
Fe^{3+}	1.3	9.6
La^{3+}	2.3	9.04
Nanochannels	Bare (nm)	Gold deposition (nm)
Polycarbonate membrane	20	1.8

Table S3. The pH data for these two electrolyte solutions (KCl and $LaCl_3$) with varying concentrations

pH (25 ± 0.5 °C)		
10^{-5} M KCl	10^{-4} M KCl	10^{-3} M KCl
6.05	6.10	6.00
10^{-2} M KCl	10^{-1} M KCl	1 M KCl
6.14	6.04	5.99
10^{-5} M $LaCl_3$	10^{-4} M $LaCl_3$	10^{-3} M $LaCl_3$
5.70	5.54	5.71
10^{-2} M $LaCl_3$	10^{-1} M $LaCl_3$	1 M $LaCl_3$
5.65	5.31	4.65

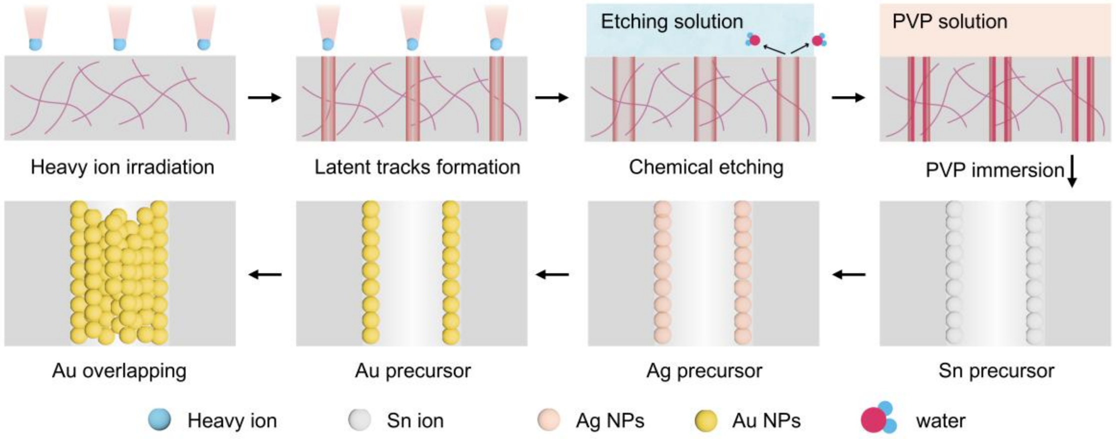

Fig. S1. Schematic diagram of fabrication process for the PCTE membrane coated with electroless deposition Au NPs.

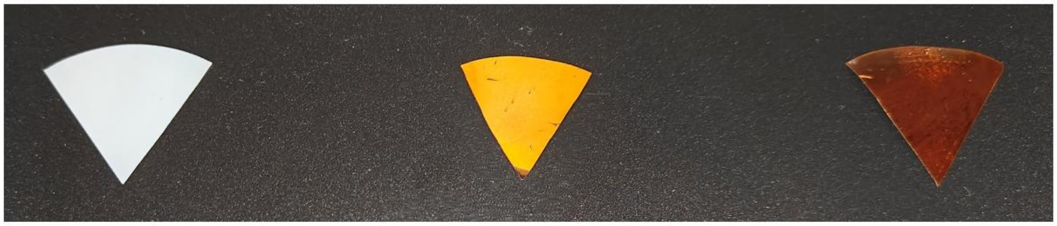

Fig. S2. The image of PC membranes used in our experiments: bare PC membrane (left), PCTE-Au membrane with outer gold layer (middle), PCTE-Au membrane without outer gold layer (right).

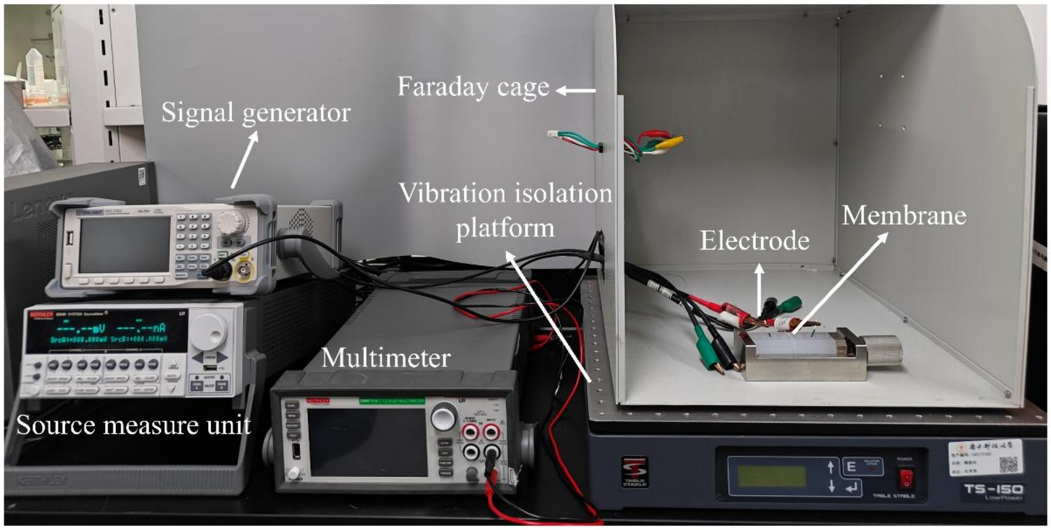

Fig. S3. The image of the electrochemical measurement setup.

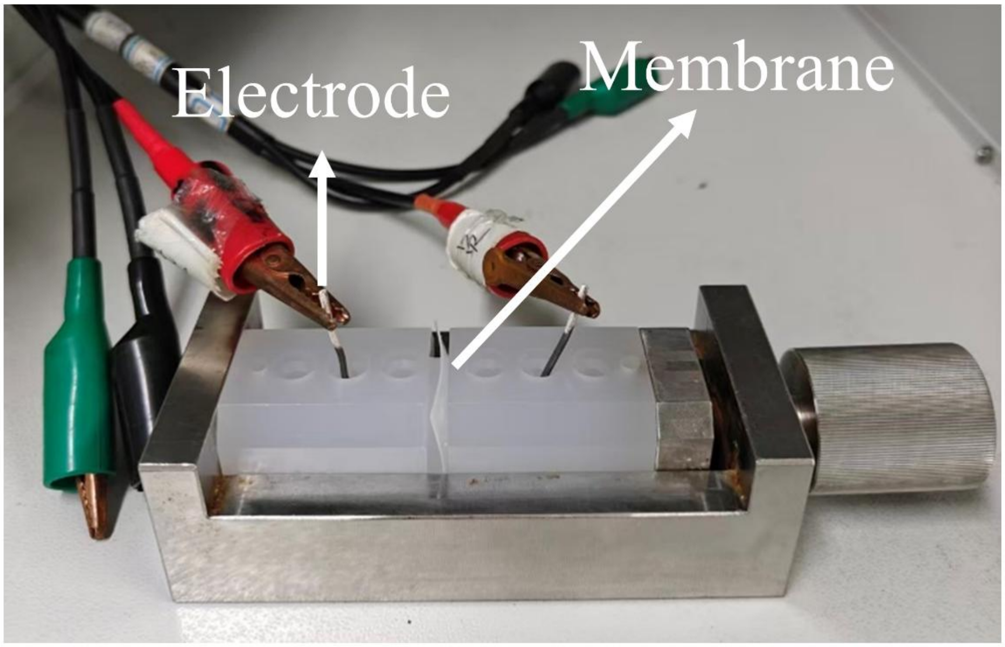

Fig. S4. The image of connection for the electrochemical *I*-*V* (or EIS) measurement setup.

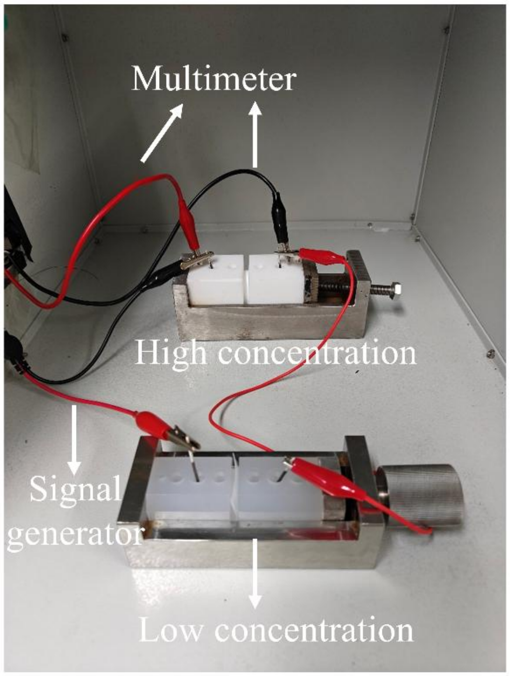

Fig. S5. The image of connection for the high pass filter circuit measurement setup.

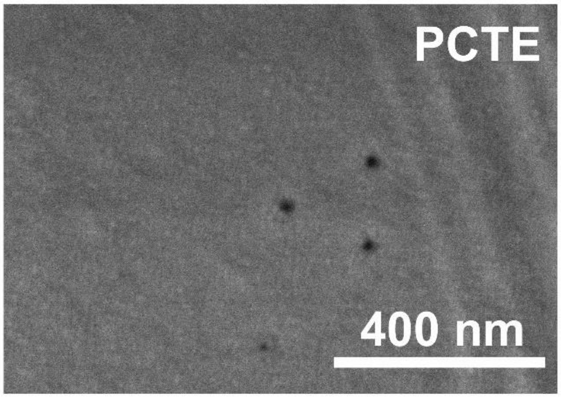

Fig. S6. SEM image of top-view 20 nm PCTE membrane.

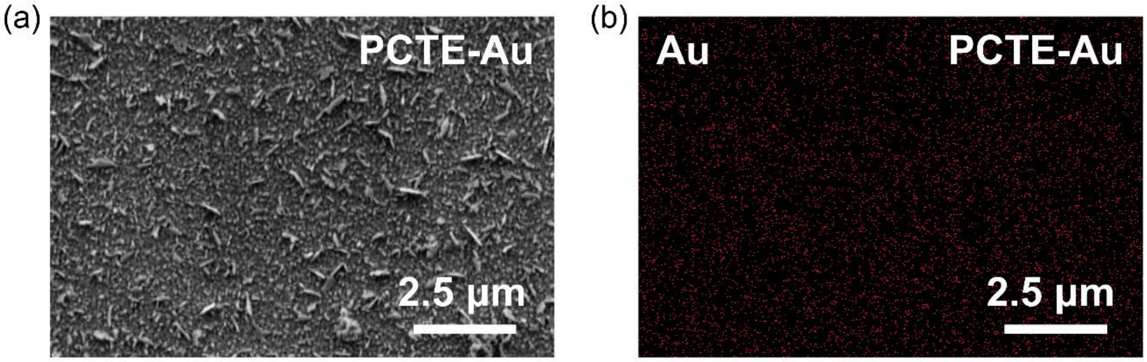

Fig. S7. Element analysis of PCTE-Au membrane. (a) SEM image of the top-view PCTE-Au membrane. (b) EDS image of top-view PCTE-Au membrane.

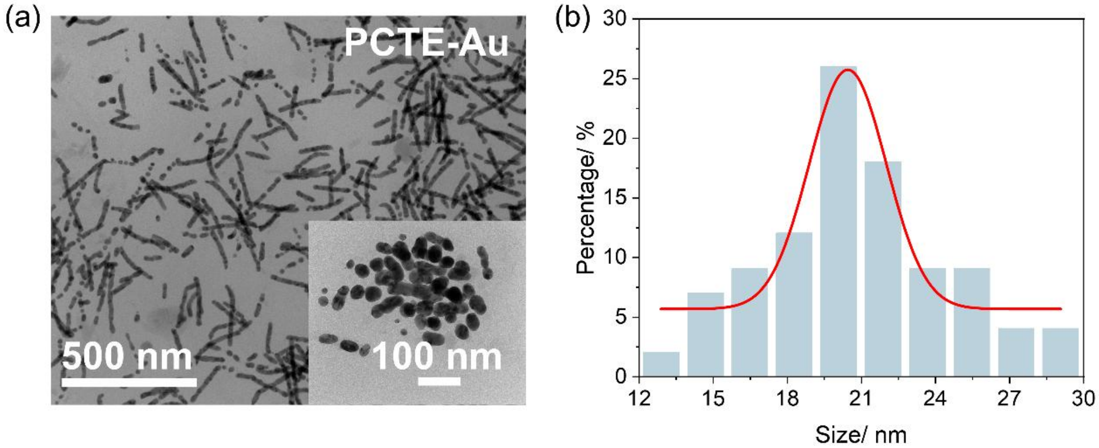

Fig. S8. Characterization of the deposited Au NPs within the PCTE-Au membrane. (a) TEM image of Au nanoparticles after dissolving PCTE-Au membrane (20 nm) in CH_2Cl_2. (b) The size distribution of Au NPs in Fig. (a).

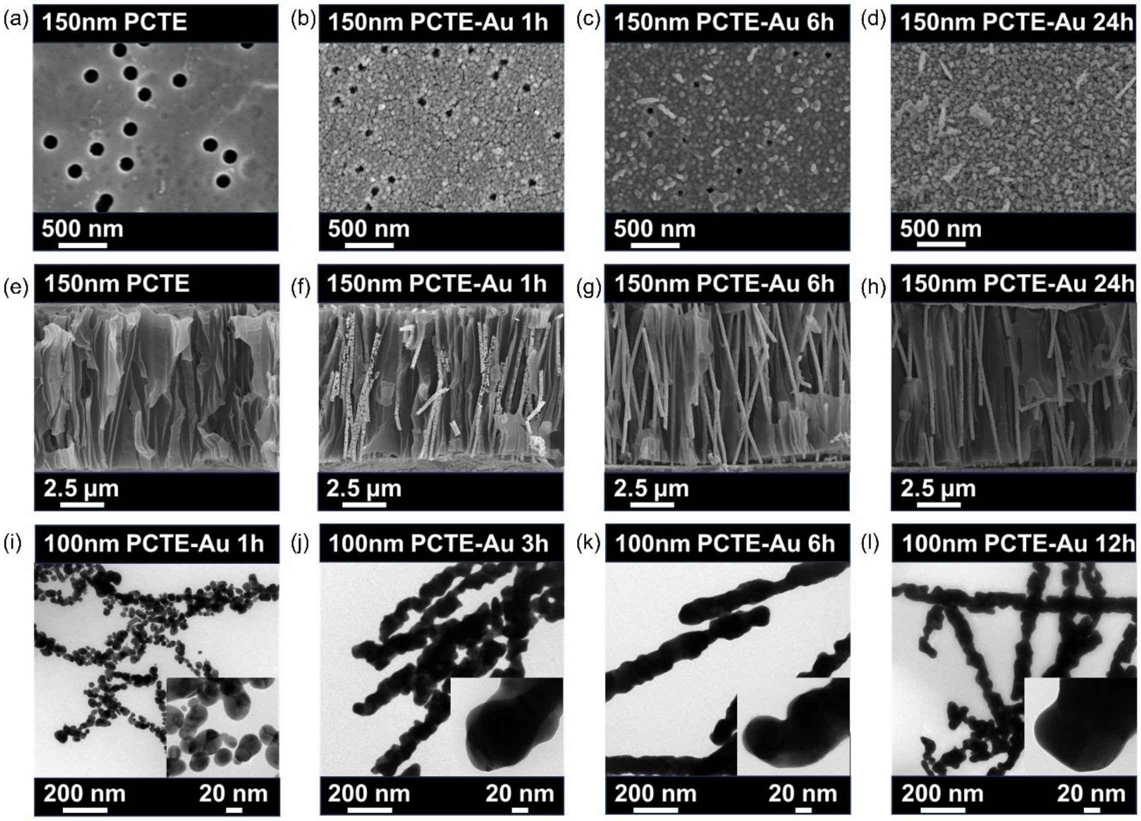

Fig. S9. Au NPs electroless deposition process in PCTE membrane. (a-d) Top-view SEM images of pristine PCTE membrane and PCTE membrane with Au electroless deposition in 1 h, 6 h and 24 h, respectively. (e-h) Side-view SEM images of pristine PCTE membrane and PCTE membrane with Au electroless deposition in 1 h, 6 h and 24 h, respectively. (i-l) TEM image of Au NPs after dissolving PCTE membrane in CH_2Cl_2.

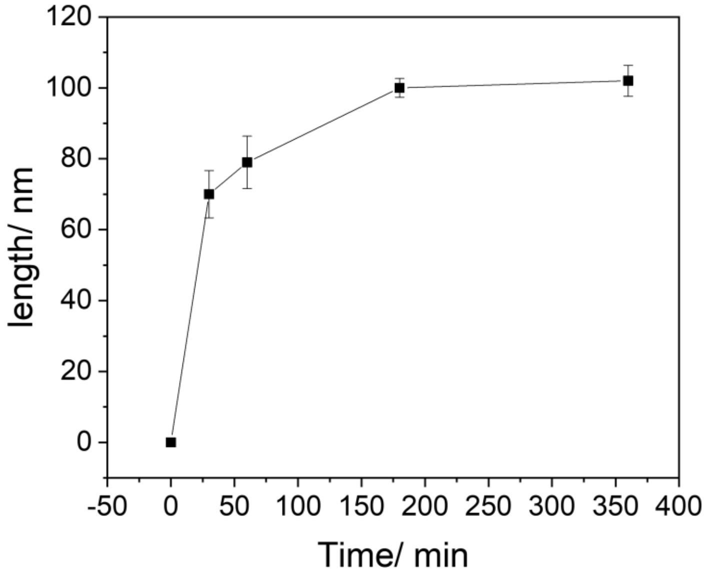

Fig. S10. Au NPs electroless deposition speed based on the SEM data.

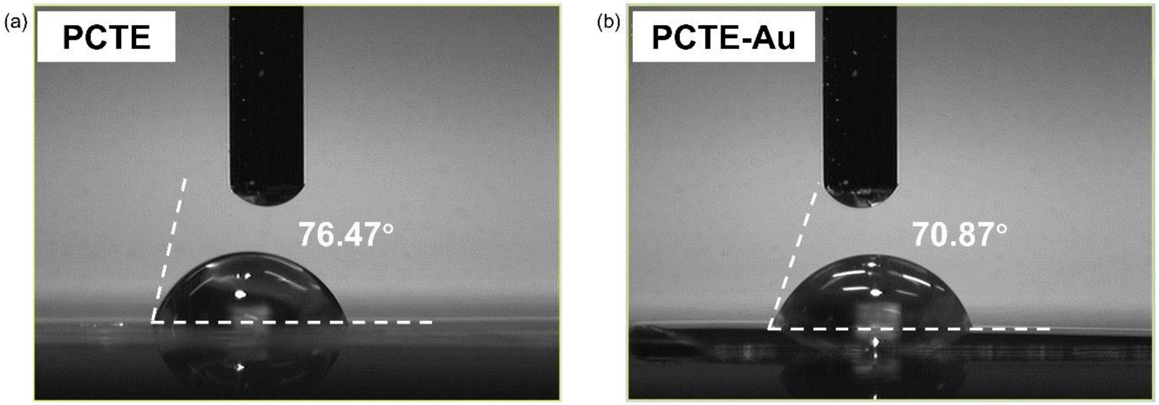

Fig. 11. The contact angle of PCTE membrane (a) before and (b) after Au electroless deposition.

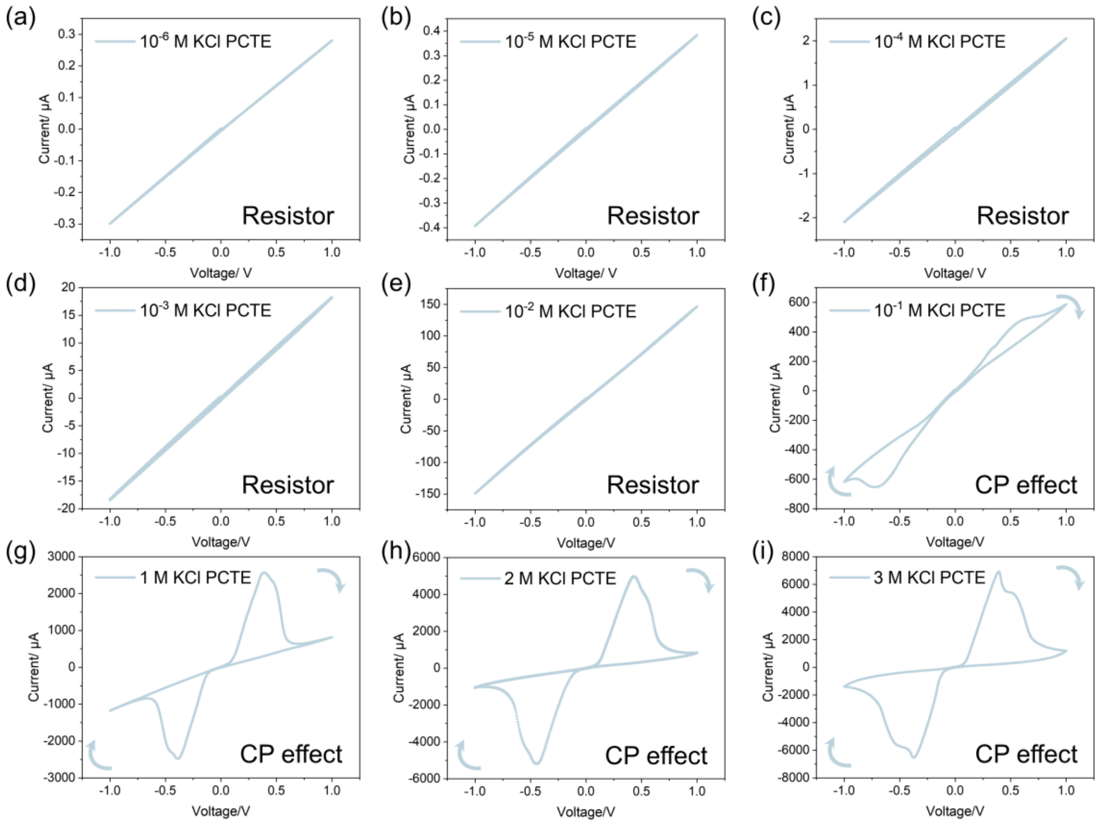

Fig. S12. Typical *I-V* curve for pristine 20 nm PCTE membrane in different concentration KCl solution. (a-i) *I-V* curve for KCl solution with concentration from 10^{-6} M to 3 M, respectively.

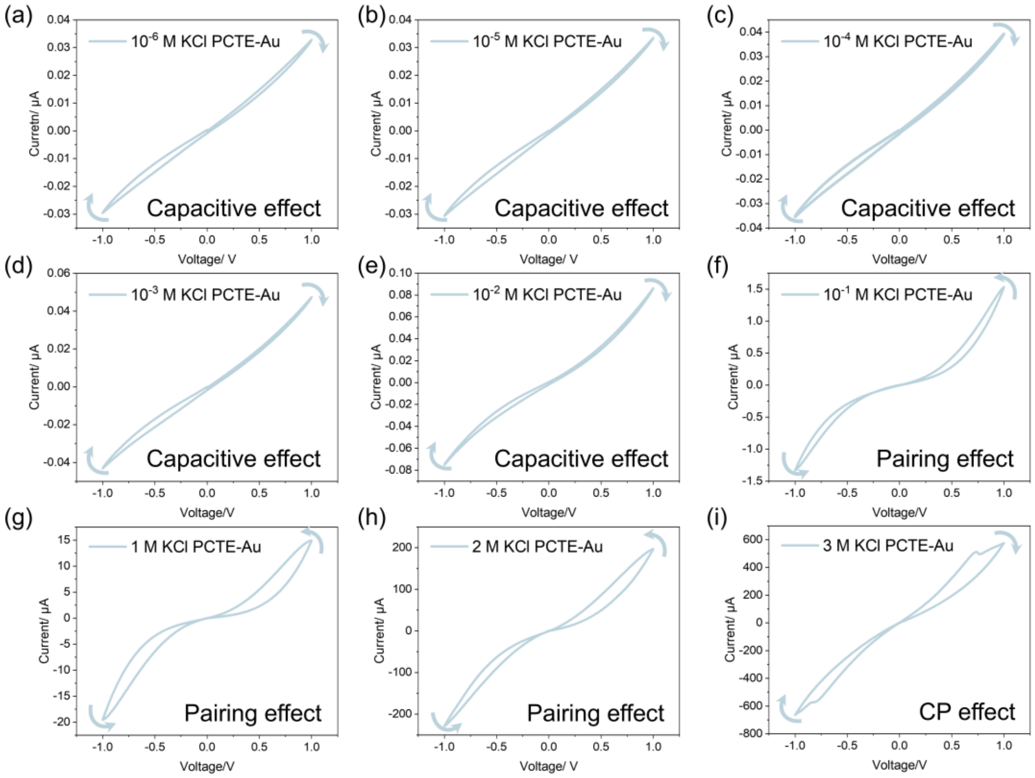

Fig. S13. Typical *I-V* curve for electroless deposition Au 20 nm PCTE membrane in different concentration KCl solution. (a-i) *I-V* curve for KCl solution with concentration from 10^{-6} M to 3 M, respectively.

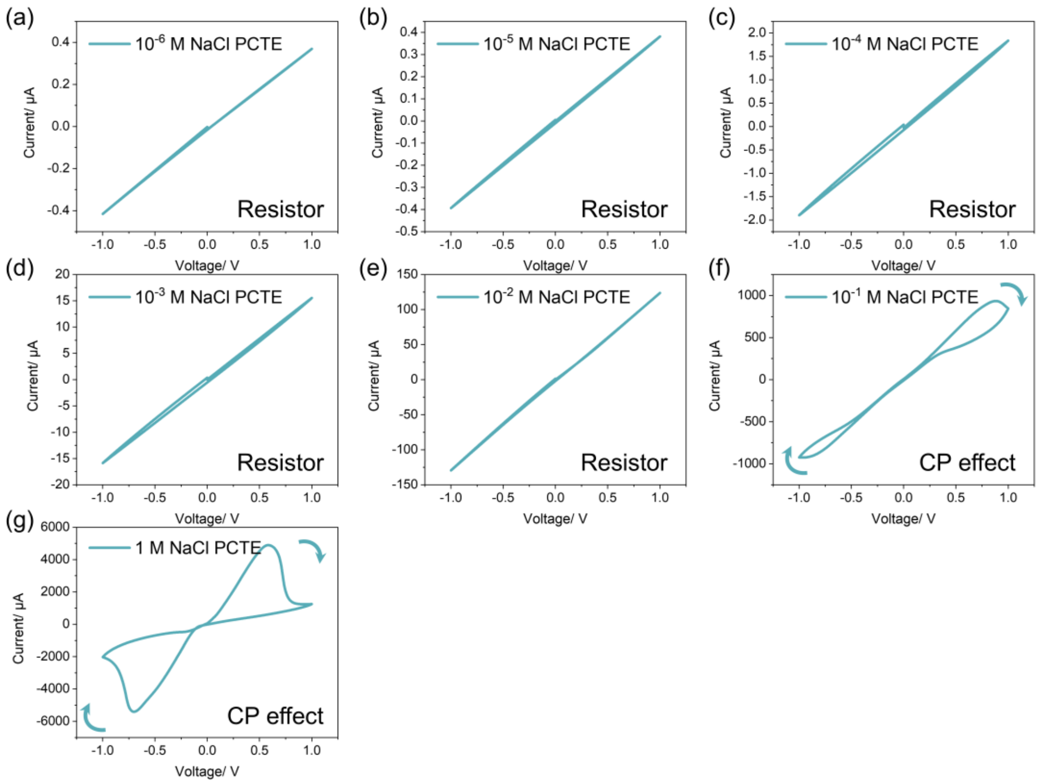

Fig. S14. Typical *I-V* curve for pristine 20 nm PCTE membrane in different concentration NaCl solution. (a-g) *I-V* curve for NaCl solution with concentration from 10^{-6} M to 1 M, respectively.

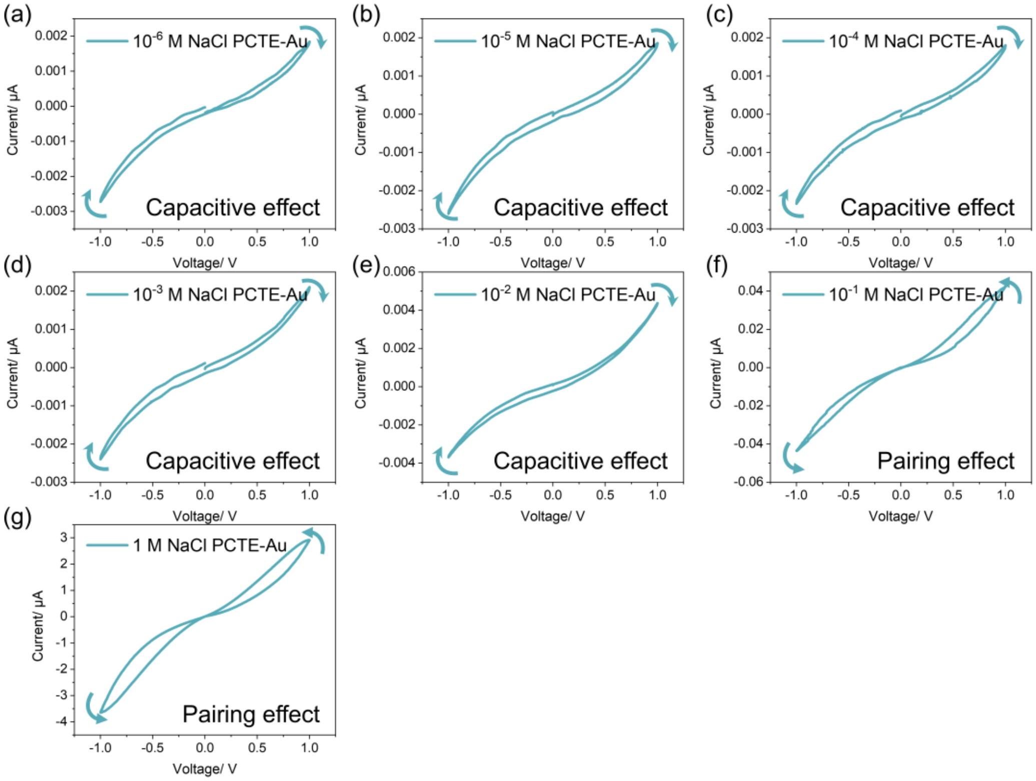

Fig. S15. Typical *I-V* curve for electroless deposition Au 20 nm PCTE membrane in different concentration NaCl solution. (a-g) *I-V* curve for NaCl solution with concentration from 10^{-6} M to 1 M, respectively.

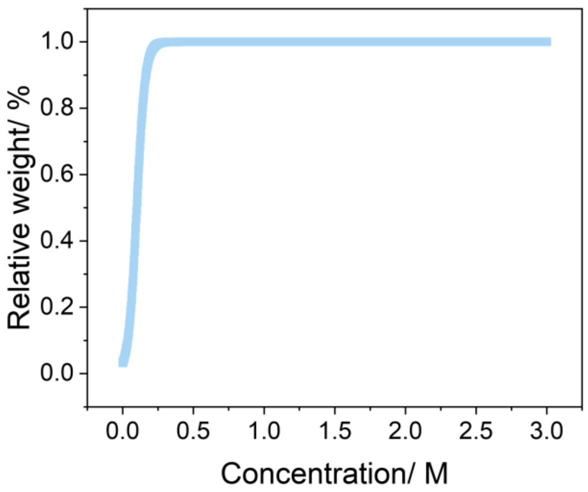

Fig. S16. The graph of weighting function $\boldsymbol{z}(\boldsymbol{c})$ showing the sharpness of the transition around the critical concentration.

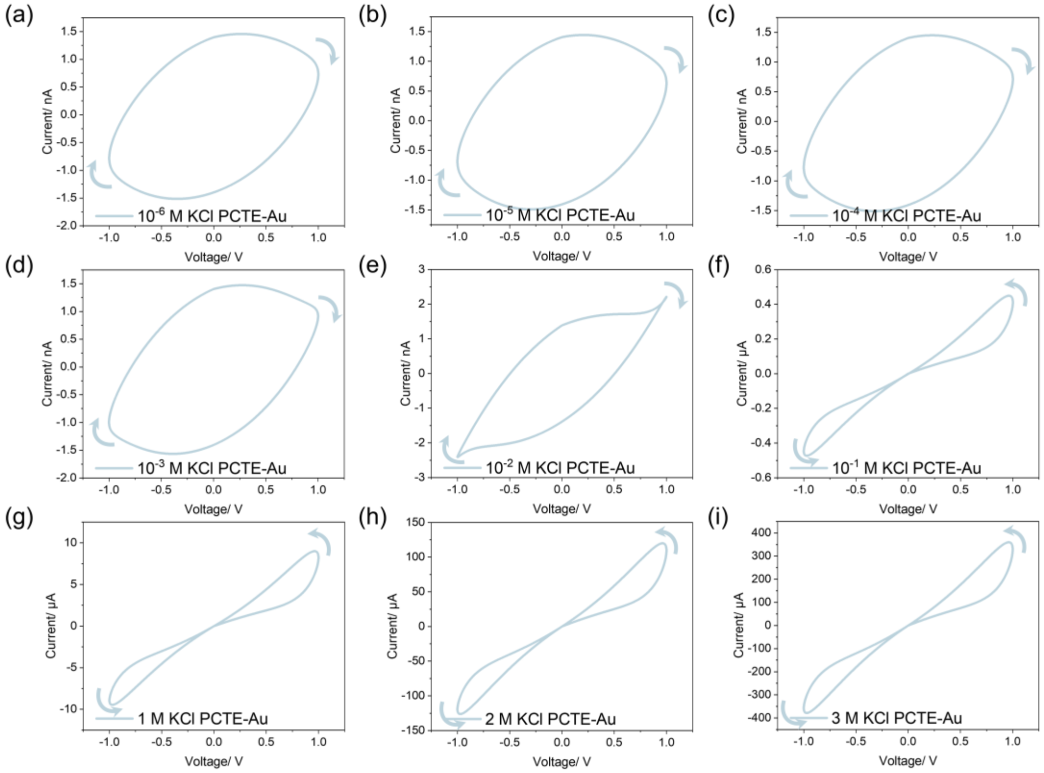

Fig. S17. Simulated typical *I-V* curve for the combined system with nanofluidic EDL effect and ion pairing effect coexisting. (a-i) *I-V* curve for KCl solution with concentration from 10^{-6} M to 3 M, respectively.

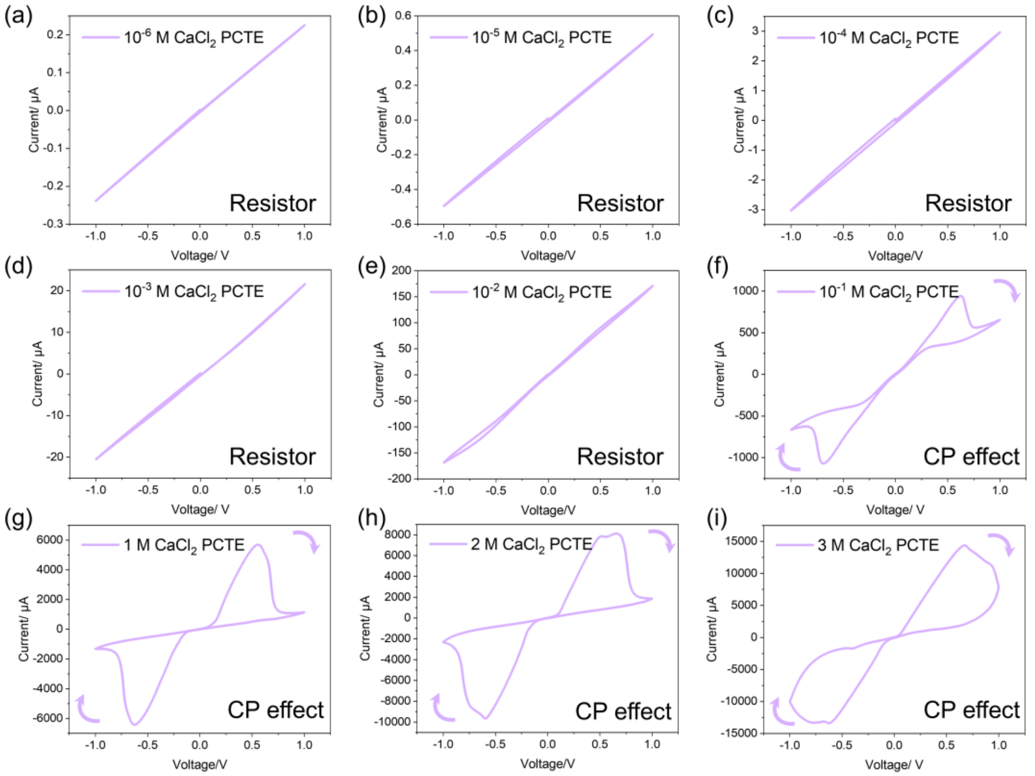

Fig. S18. Typical *I-V* curve for pristine 20 nm PCTE membrane in different concentration $CaCl_2$ solution. (a-i) *I-V* curve for $CaCl_2$ solution with concentration from 10^{-6} M to 3 M, respectively.

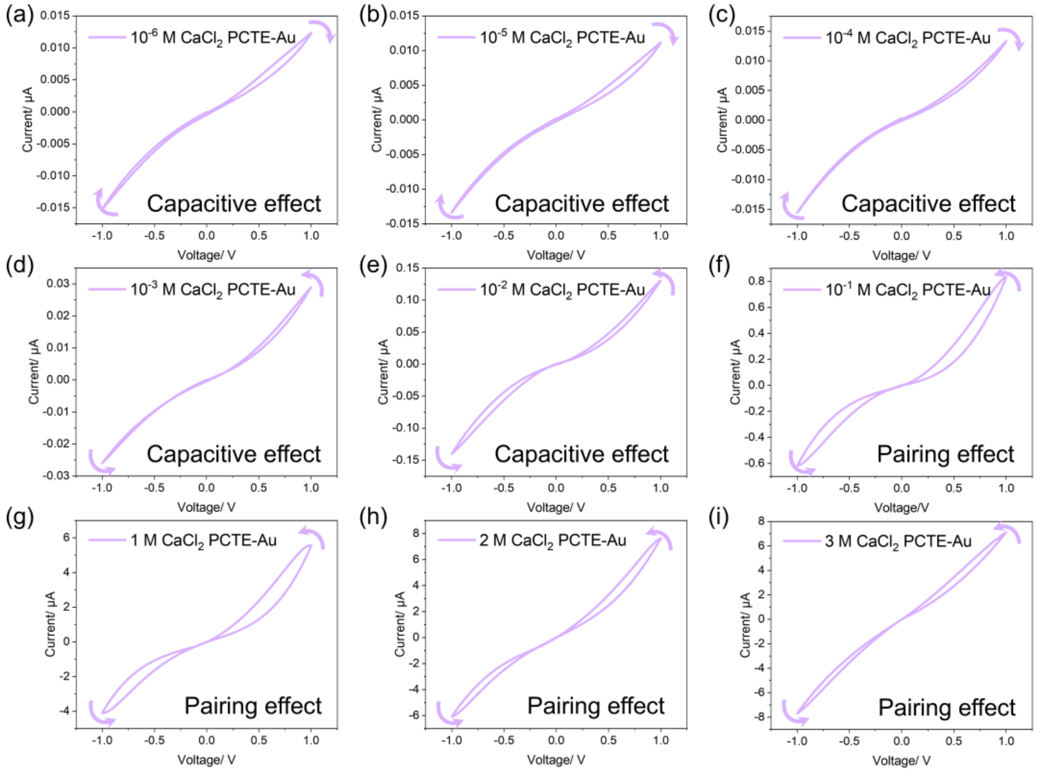

Fig. S19. Typical *I-V* curve for electroless deposition Au 20 nm PCTE membrane in different concentration $CaCl_2$ solution. (a-i) *I-V* curve for $CaCl_2$ solution with concentration from 10^{-6} M to 3 M, respectively.

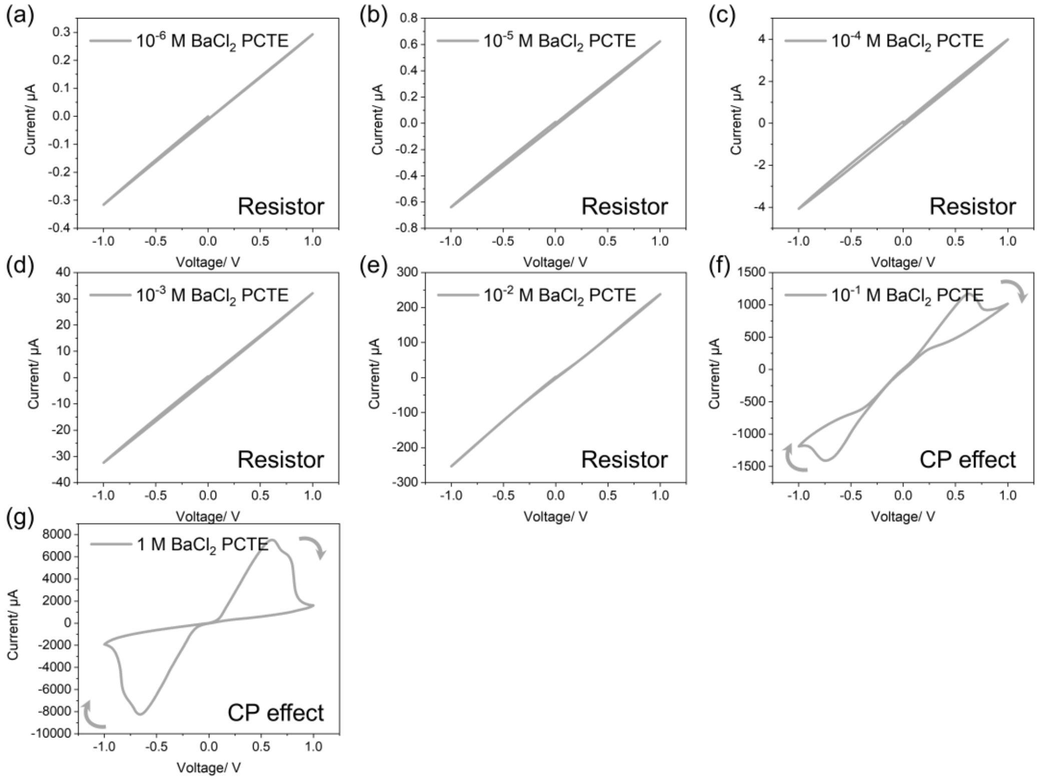

Fig. S20. Typical *I-V* curve for pristine 20 nm PCTE membrane in different concentration $BaCl_2$ solution. (a-g) *I-V* curve for $BaCl_2$ solution with concentration from 10^{-6} M to 1 M, respectively.

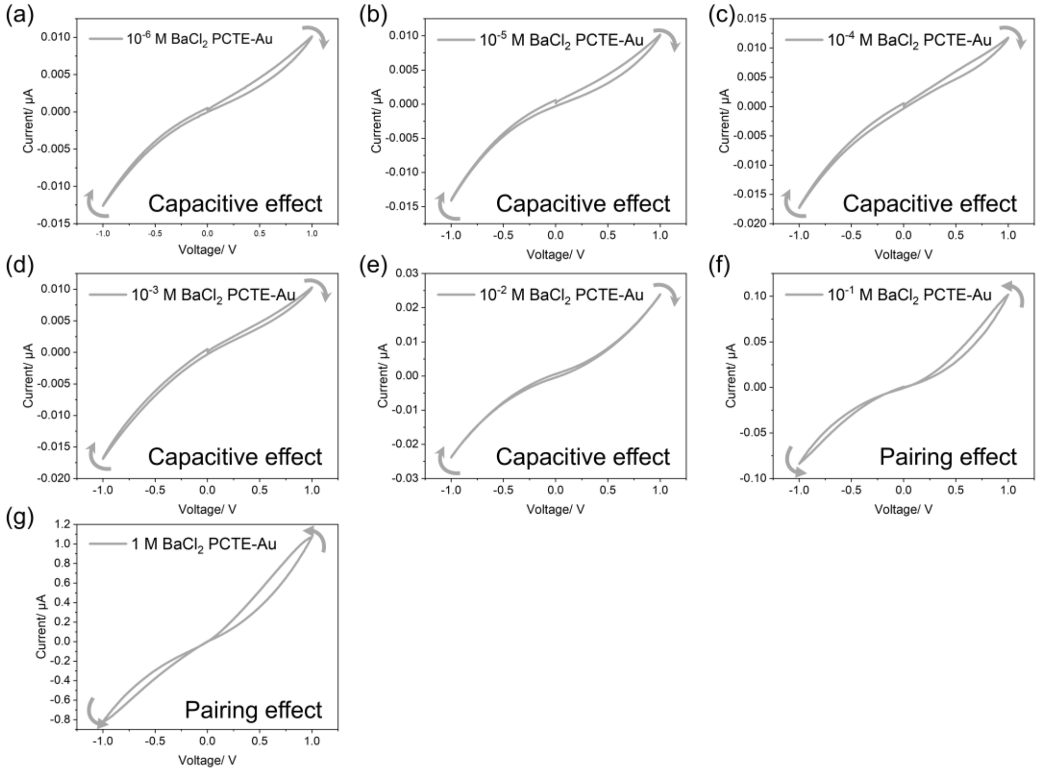

Fig. S21. Typical *I*-*V* curve for electroless deposition Au 20 nm PCTE membrane in different concentration $BaCl_2$ solution. (a-g) *I*-*V* curve for $BaCl_2$ solution with concentration from 10^{-6} M to 1 M, respectively.

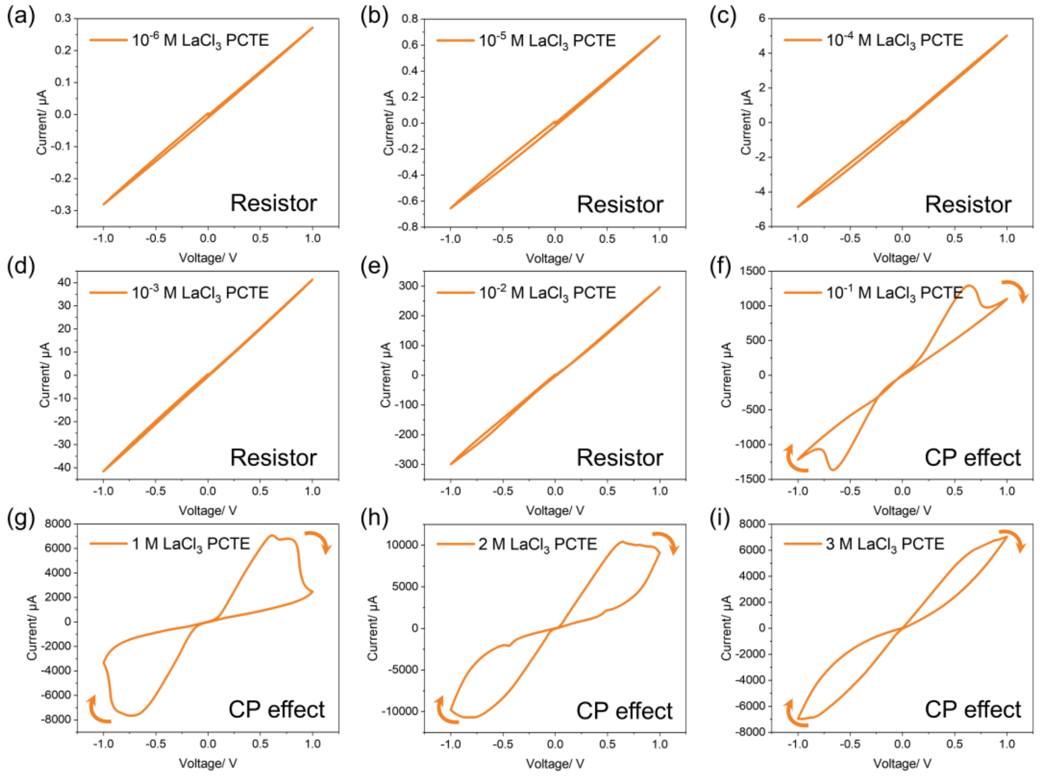

Fig. S22. Typical *I-V* curve for pristine 20 nm PCTE membrane in different concentration $LaCl_3$ solution. (a-i) *I-V* curve for $LaCl_3$ solution with concentration from 10^{-6} M to 3 M, respectively.

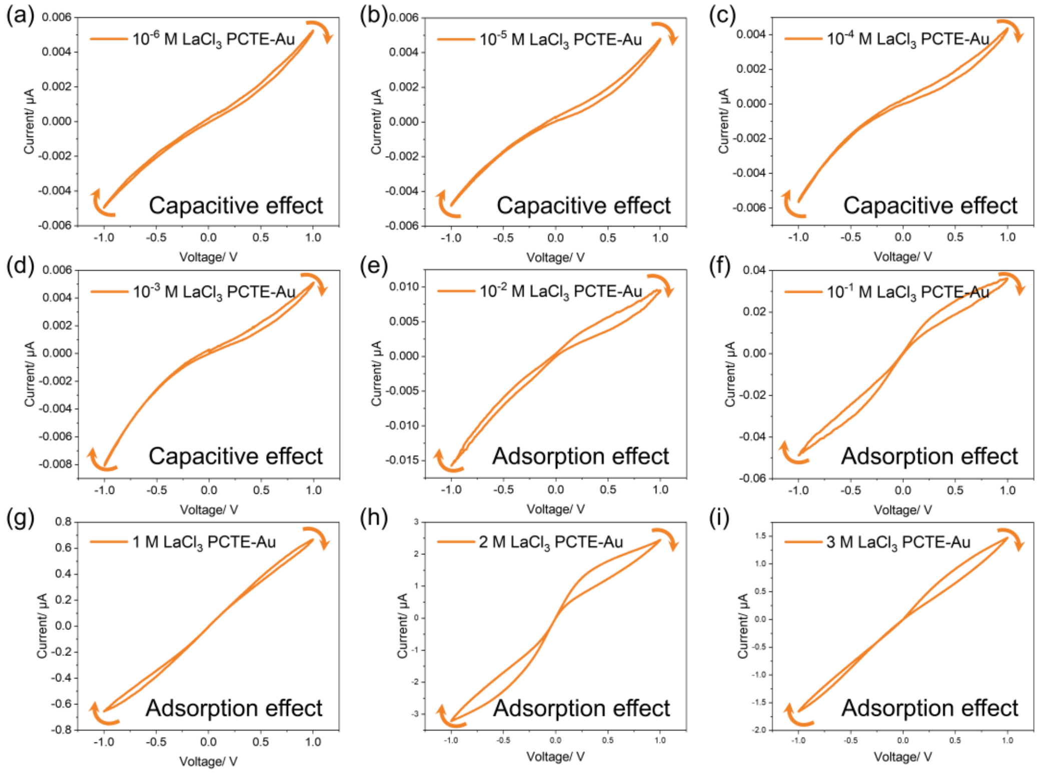

Fig. S23. Typical *I-V* curve for electroless deposition Au 20 nm PCTE membrane in different concentration $LaCl_3$ solution. (a-i) *I-V* curve for $LaCl_3$ solution with concentration from 10^{-6} M to 3 M, respectively.

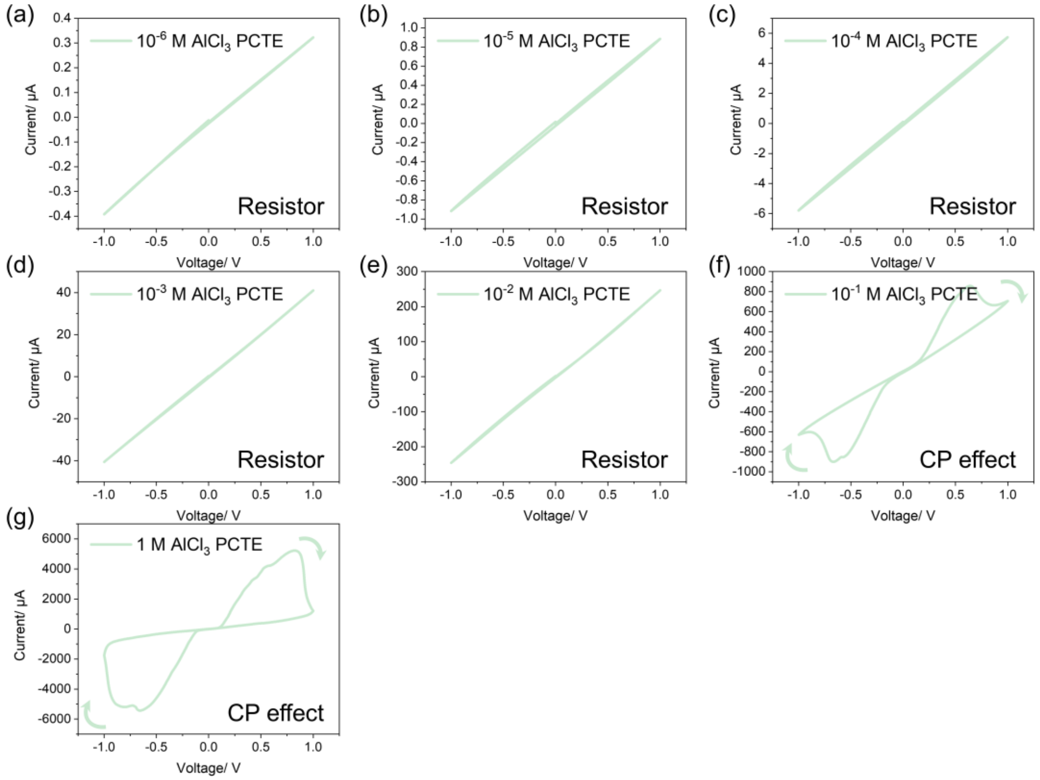

Fig. S24. Typical *I-V* curve for pristine 20 nm PCTE membrane in different concentration $AlCl_3$ solution. (a-g) *I-V* curve for $AlCl_3$ solution with concentration from 10^{-6} M to 1 M, respectively.

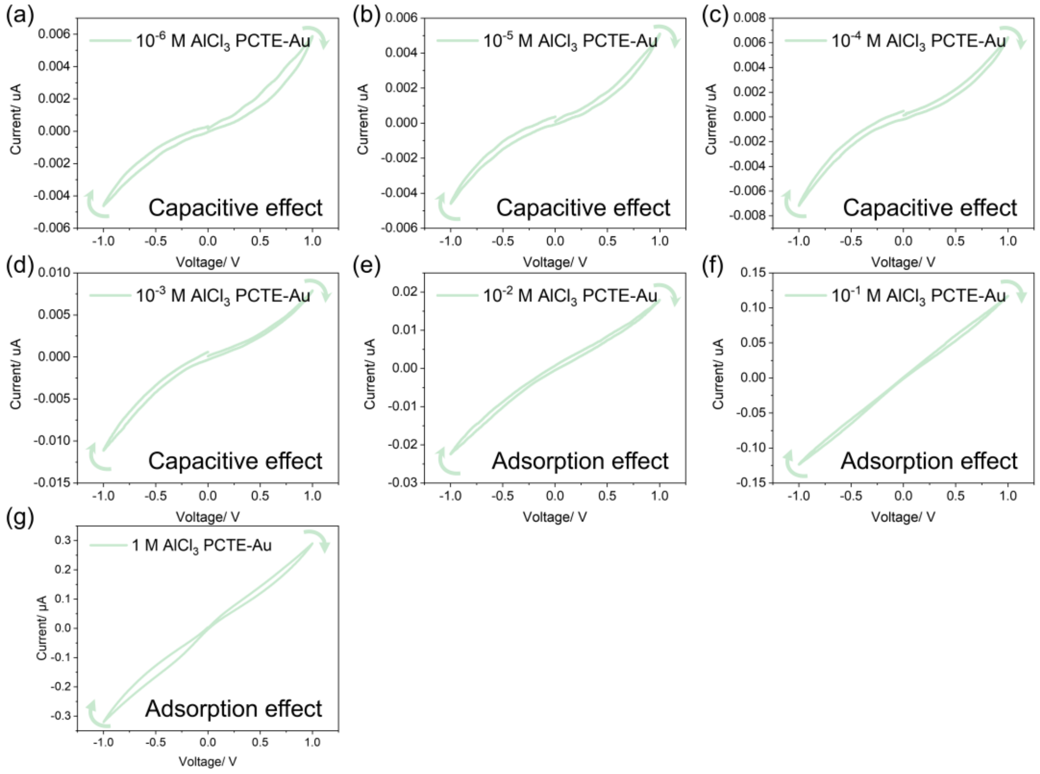

Fig. S25. Typical *I-V* curve for electroless deposition Au 20 nm PCTE membrane in different concentration $AlCl_3$ solution. (a-g) *I-V* curve for $AlCl_3$ solution with concentration from 10^{-6} M to 1 M, respectively.

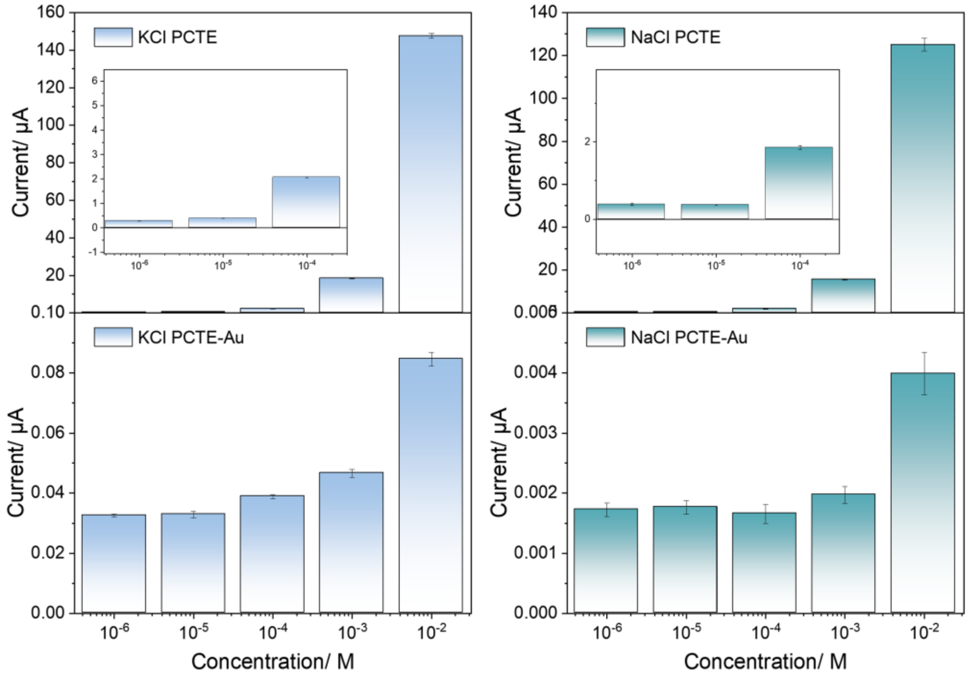

Fig. S26. Current change before and after Au NPs deposition for KCl and NaCl solutions.

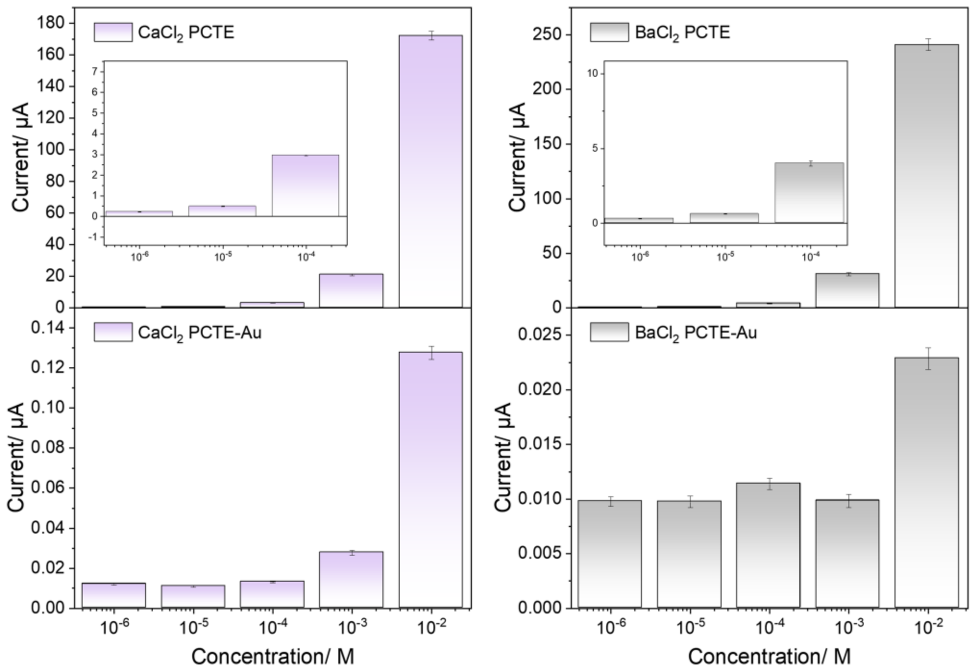

Fig. S27. Current change before and after Au NPs deposition for $CaCl_2$ and $BaCl_2$ solutions.

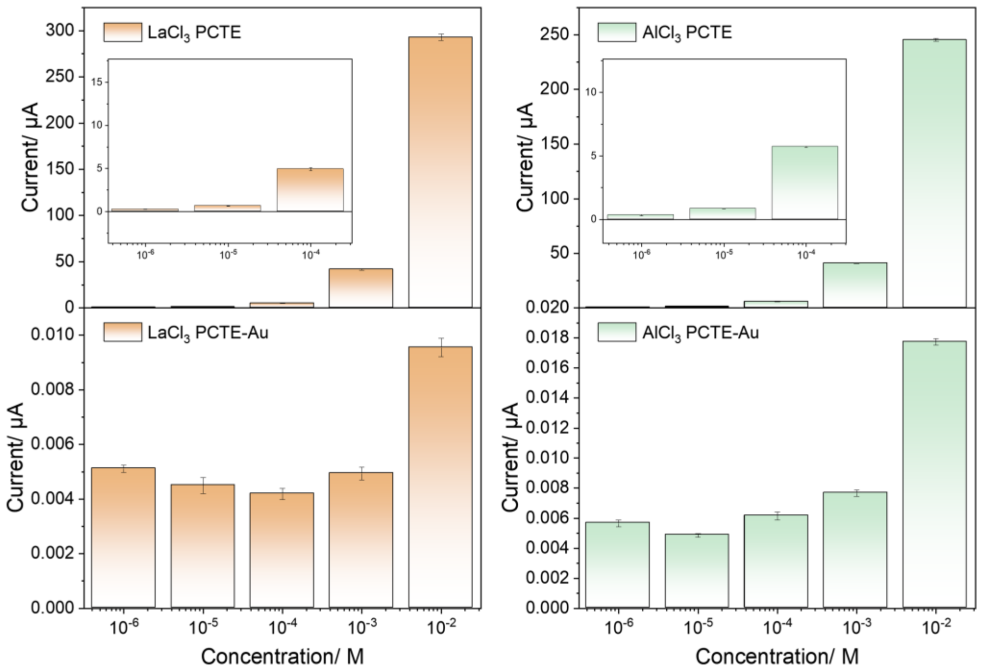

Fig. S28. Current change before and after Au NPs deposition for $LaCl_3$ and $AlCl_3$ solutions.

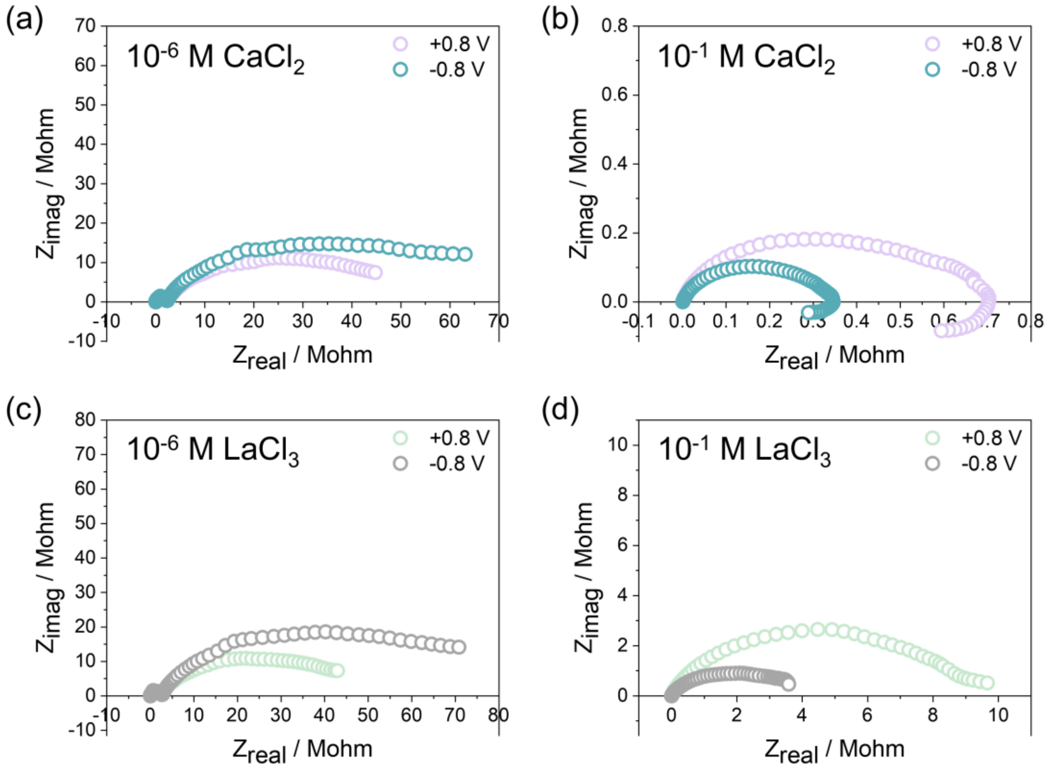

Fig. S29. EIS of 20 nm PCTE-Au membrane in (a) 10^{-6} M $CaCl_2$, (b) 10^{-1} M $CaCl_2$, (c) 10^{-6} M $LaCl_3$ and (d) 10^{-1} M $LaCl_3$ solutions, respectively.

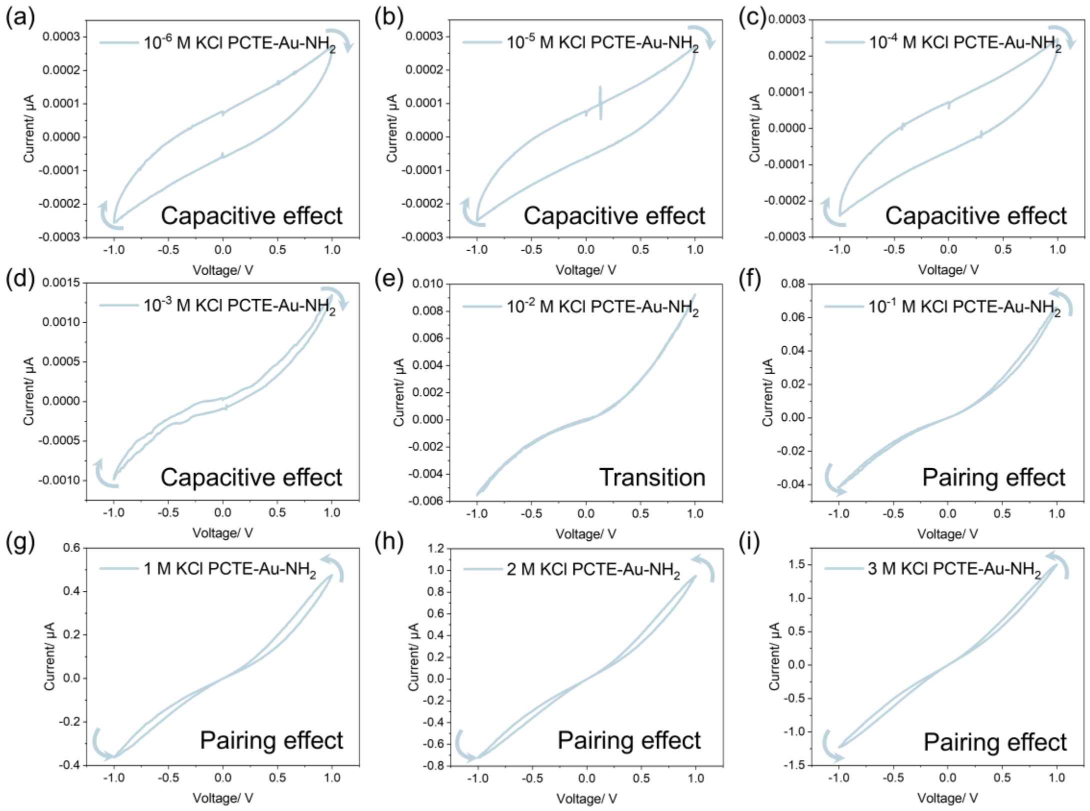

Fig. S30. Typical *I*-*V* curve for cysteamine decorated electroless deposition Au 20 nm PCTE membrane in different concentration KCl solution. (a-i) *I*-*V* curve for KCl solution with concentration from 10^{-6} M to 3 M, respectively.

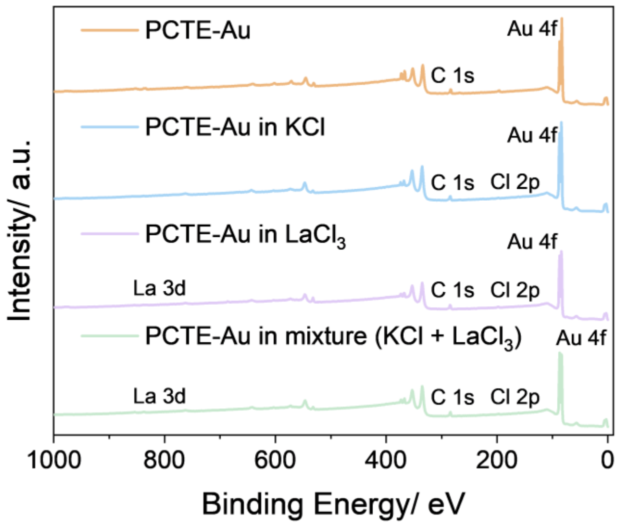

Fig. S31. XPS survey data of four PCTE-Au membranes in different solutions (purified water; KCl solution; $LaCl_3$ solution; an equal-volume (1 : 1, v/v) mixture of KCl and $LaCl_3$ solutions).

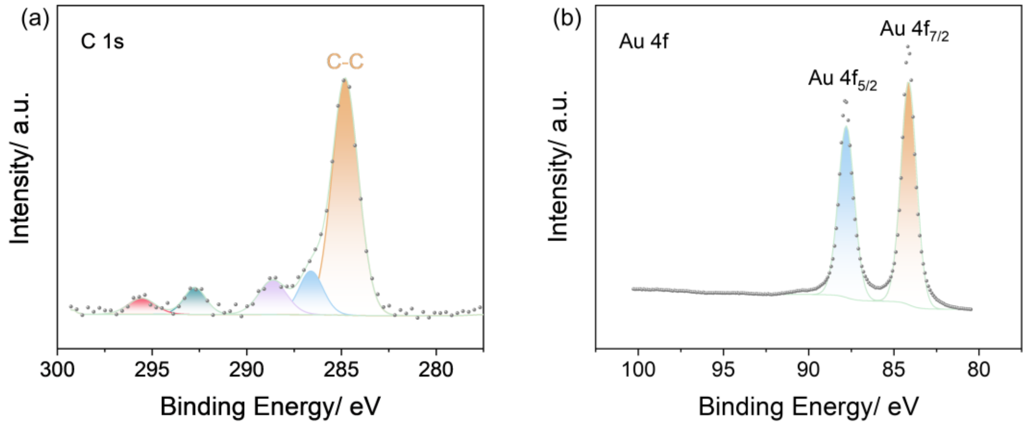

Fig. S32. High-resolution XPS spectra for the PCTE-Au membrane in purified water.

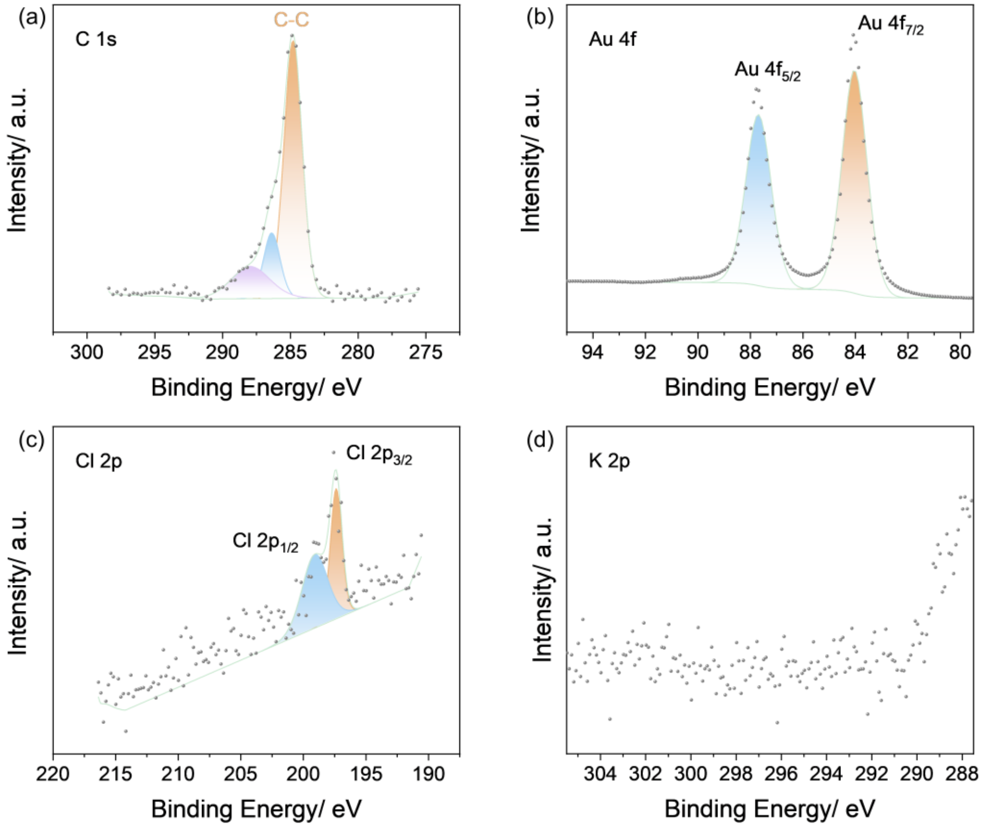

Fig. S33. High-resolution XPS spectra for the PCTE-Au membrane in KCl solution.

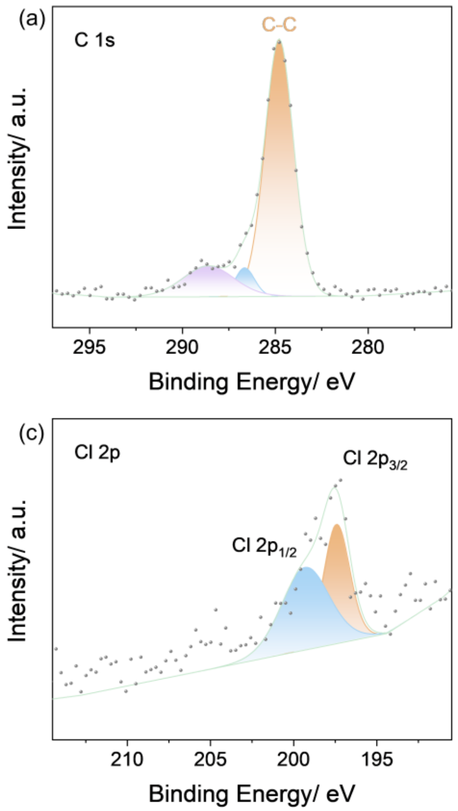

Fig. S34. High-resolution XPS spectra for the PCTE-Au membrane in $LaCl_3$ solution.

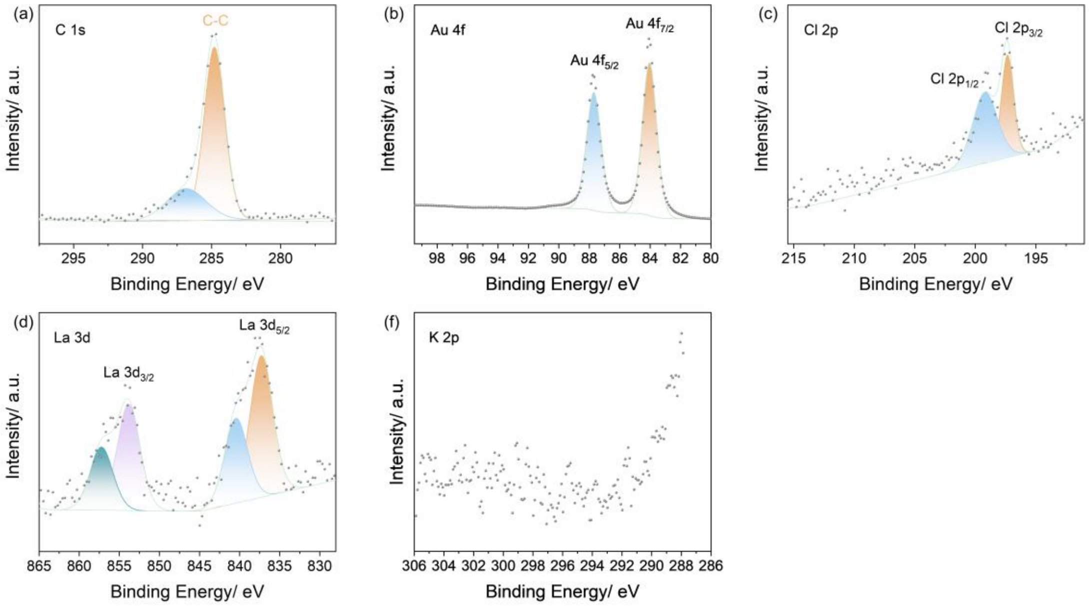

Fig. S35. High-resolution XPS spectra for the PCTE-Au membrane in equal-volume (1 : 1, v/v) mixture of KCl and $LaCl_3$ solutions.

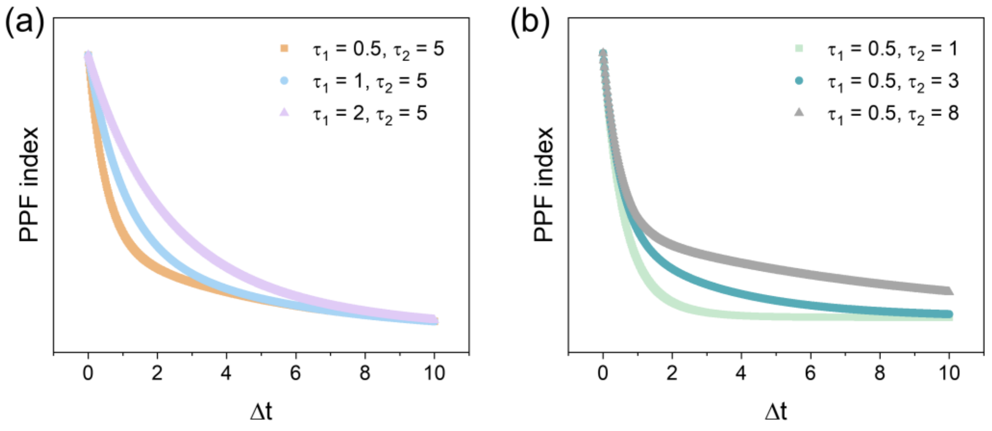

Fig. S36. The effect of the time constant variation on the device performance.

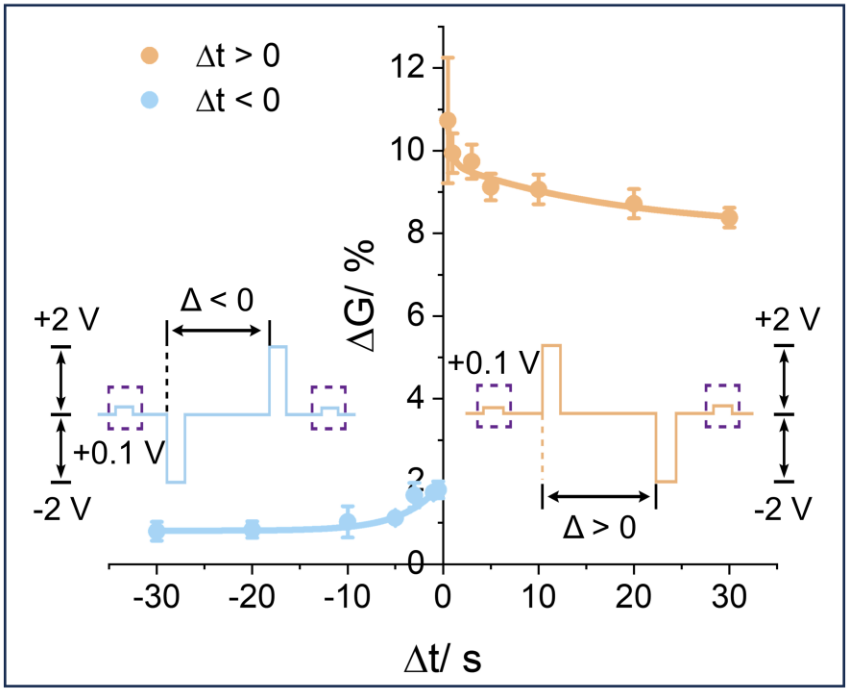

Fig. S37. The plot of the conductance change with variation in Δt showing the STDP response.

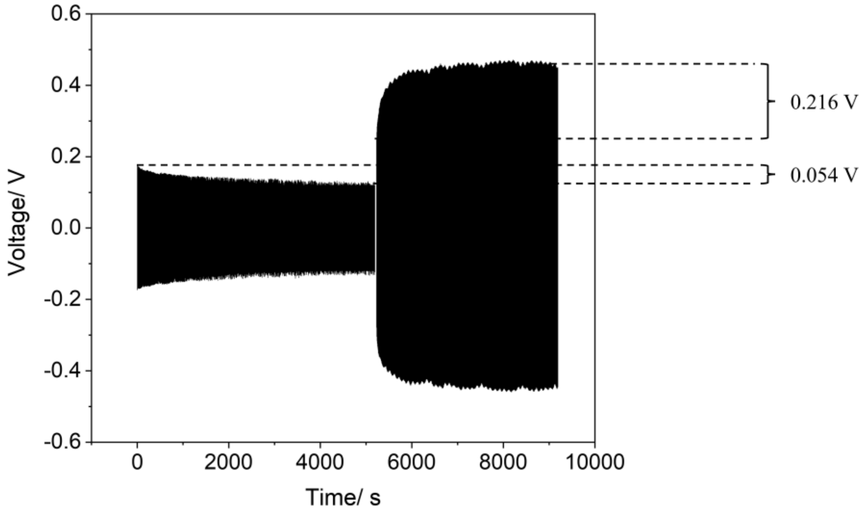

Fig. S38. The amplitude change of the 120 Hz signal in the high-pass filter circuit.

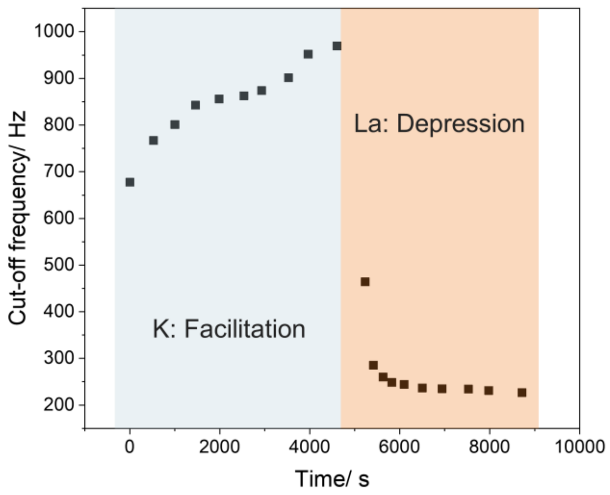

Fig. S39. The varying cut-off frequency of our high-pass filter circuit for both electrolytes.

SI References

1. Esquivel-Sirvent R. Anomaly of the dielectric function of water under confinement and its role in van der Waals interactions. *Phys Rev E* 2020; **102**: 042609.
2. Fumagalli L, Esfandiar A, Fabregas R *et al.* Anomalously low dielectric constant of confined water. *Science* 2018; **360**: 1339-42.
3. Hansen J-P and McDonald IR. Theory of simple liquids: with applications to soft matter: Academic press, 2013.
4. Brüesch P and Christen T. The electric double layer at a metal electrode in pure water. *J Appl Phys* 2004; **95**: 2846-56.
5. Janz GJ and Tomkins RPT. Conductance Cell Calibrations: Current Practices. *Journal of The Electrochemical Society* 1977; **124**: 55C.
6. Robin P, Emmerich T, Ismail A *et al.* Long-term memory and synapse-like dynamics in two-dimensional nanofluidic channels. *Science* 2023; **379**: 161-7.
7. Campbell GA. Physical theory of the electric wave-filter. *Bell System Tech Jol* 1922; **1**: 1-32.
8. Ravikovitch PI, Vishnyakov A, Russo R *et al.* Unified Approach to Pore Size Characterization of Microporous Carbonaceous Materials from N2, Ar, and CO2 Adsorption Isotherms. *Langmuir* 2000; **16**: 2311-20.
9. Thommes M, Kaneko K, Neimark AV *et al.* Physisorption of gases, with special reference to the evaluation of surface area and pore size distribution (IUPAC Technical Report). 2015; **87**: 1051-69.
10. Landers J, Gor GY, Neimark AV. Density functional theory methods for characterization of porous materials. *Colloids Surf A Physicochem Eng Asp* 2013; **437**: 3-32.
